# Comparative Genomics of *Gardnerella vaginalis* Strains Reveals Substantial Differences in Metabolic and Virulence Potential

**DOI:** 10.1371/journal.pone.0012411

**Published:** 2010-08-26

**Authors:** Carl J. Yeoman, Suleyman Yildirim, Susan M. Thomas, A. Scott Durkin, Manolito Torralba, Granger Sutton, Christian J. Buhay, Yan Ding, Shannon P. Dugan-Rocha, Donna M. Muzny, Xiang Qin, Richard A. Gibbs, Steven R. Leigh, Rebecca Stumpf, Bryan A. White, Sarah K. Highlander, Karen E. Nelson, Brenda A. Wilson

**Affiliations:** 1 Institute for Genomic Biology, University of Illinois, Urbana, Illinois, United States of America; 2 J. Craig Venter Institute, Rockville, Maryland, United States of America; 3 Human Genome Sequencing Center, Baylor College of Medicine, Houston, Texas, United States of America; 4 Department of Anthropology, University of Illinois, Urbana, Illinois, United States of America; 5 Department of Animal Sciences, University of Illinois, Urbana, Illinois, United States of America; 6 Department of Molecular Virology and Microbiology, Baylor College of Medicine, Houston, Texas, United States of America; 7 Department of Microbiology, University of Illinois, Urbana, Illinois, United States of America; Duke University Medical Center, United States of America

## Abstract

**Background:**

*Gardnerella vaginalis* is described as a common vaginal bacterial species whose presence correlates strongly with bacterial vaginosis (BV). Here we report the genome sequencing and comparative analyses of three strains of *G. vaginalis*. Strains 317 (ATCC 14019) and 594 (ATCC 14018) were isolated from the vaginal tracts of women with symptomatic BV, while Strain 409-05 was isolated from a healthy, asymptomatic individual with a Nugent score of 9.

**Principal Findings:**

Substantial genomic rearrangement and heterogeneity were observed that appeared to have resulted from both mobile elements and substantial lateral gene transfer. These genomic differences translated to differences in metabolic potential. All strains are equipped with significant virulence potential, including genes encoding the previously described vaginolysin, pili for cytoadhesion, EPS biosynthetic genes for biofilm formation, and antimicrobial resistance systems, We also observed systems promoting multi-drug and lantibiotic extrusion. All *G. vaginalis* strains possess a large number of genes that may enhance their ability to compete with and exclude other vaginal colonists. These include up to six toxin-antitoxin systems and up to nine additional antitoxins lacking cognate toxins, several of which are clustered within each genome. All strains encode bacteriocidal toxins, including two lysozyme-like toxins produced uniquely by strain 409-05. Interestingly, the BV isolates encode numerous proteins not found in strain 409-05 that likely increase their pathogenic potential. These include enzymes enabling mucin degradation, a trait previously described to strongly correlate with BV, although commonly attributed to non-*G. vaginalis* species.

**Conclusions:**

Collectively, our results indicate that all three strains are able to thrive in vaginal environments, and therein the BV isolates are capable of occupying a niche that is unique from 409-05. Each strain has significant virulence potential, although genomic and metabolic differences, such as the ability to degrade mucin, indicate that the detection of *G. vaginalis* in the vaginal tract provides only partial information on the physiological potential of the organism.

## Introduction

*Gardnerella vaginalis* is a facultatively anaerobic bacterium of the *Bifidobacteriaceae* family. *G. vaginalis* is often described as a Gram-variable organism but has a Gram-positive wall type [1]. Although *G. vaginalis* commonly occurs in the vaginal microbiota of healthy individuals [2]–[4], it has been identified as one of the frequent and predominant colonists of the vaginal tract in women diagnosed with bacterial vaginosis (BV) [2], [5], [6]. The presence of high numbers of *G. vaginalis* also correlates with both infertility and pre-term labor [5], [7]. Moreover, BV may increase the risk of sexually transmitted diseases including HIV [8]. Although the etiology of BV with respect to *G. vaginalis* remains poorly understood, *G. vaginalis* has been identified in 95% of clinically diagnosed cases. Recent research indicates that *G. vaginalis* may be more virulent than other organisms commonly associated with the disease [6]. In addition to BV, *G. vaginalis* has also been linked to vertebral osteomyelitis [9], retinal vasculitis [10] and acute hip arthritis [11]. Consequently, *G. vaginalis* is of significant interest to both clinicians and researchers.

Several strains of *G. vaginalis* have been targeted for genome sequencing as part of the National Institutes of Health (NIH)-funded Human Microbiome Project (HMP). For two of these strains, 317 (ATCC 14019) and 409-05, genome sequencing has been completed, while a draft sequence is available for a third, 594 (ATCC 14018). Strains 317 and 594 were isolated from vaginal secretions of women suffering from BV [12]. Strain 409-05 was isolated from a healthy, asymptomatic individual with a Nugent score of 9 [4], which is indicative of a BV state [13]. Further investigation of the microbiome from this individual revealed an enriched population of *G. vaginalis* (25% of the microbiome) and a reduction in the lactobacilli (20%); lactobacilli typically account for 70–90% of the microbiome in a healthy individual [4]. Here we report the comparative genomic analyses of the two finished genomes, making comparisons, where possible, to the draft genome of strain 594. We define and contrast their potential virulence features, and provide much needed genomic insights into the metabolic potentials and implicated lifestyle of *G. vaginalis*. These analyses show potentially significant differences among closely-related strains along many important dimensions, raising critical questions about bacterial pathogenesis and evolution.

## Results and Discussion

### Phylogenetic analysis

**Figure 1:**
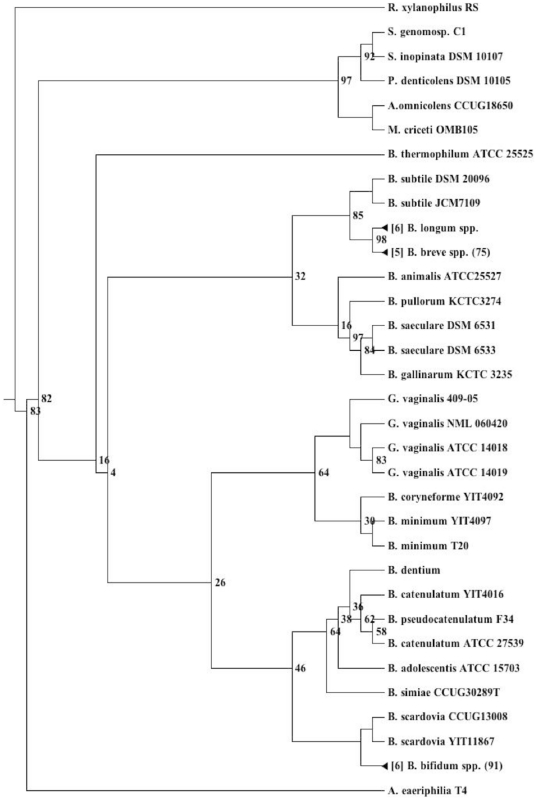
*Bifidobacteriaceae* phylogeny. Maximum likelihood 16*S* rDNA based phylogentic reconstruction of the *Bifidobacteriaceae*. Bootstrap values less than 100 are shown at each node. Larger taxonomic clusters have been collapsed for clarity.

The genus *Gardnerella* comprises a single species, *G. vaginalis*. Phylogenetic reconstruction of the 16*S* rDNA shows *G. vaginalis* forms a distinct clade within the *Bifidobacteraceae* family most closely related to *Bifidobacterium coryneforme* and *B. minimum* (Fig. 1). The 16*S* rDNA of the two *G. vaginalis* strains isolated from BV patients appear to be very similar, differing by just a single nucleotide. On the other hand strain 409-05 appears to be deeper rooting and shares only 98% 16*S* rDNA identity, close to the maximum 16*S* rDNA*-*dissimilarity commonly tolerated for strains of a single species. Given the 16*S* rDNA difference between strain 409-05 and the other two strains, comparison of their genomes therefore provides a useful indicator of the potential genetic variability within *G. vaginalis* species.

### General genome features

**Figure 2:**
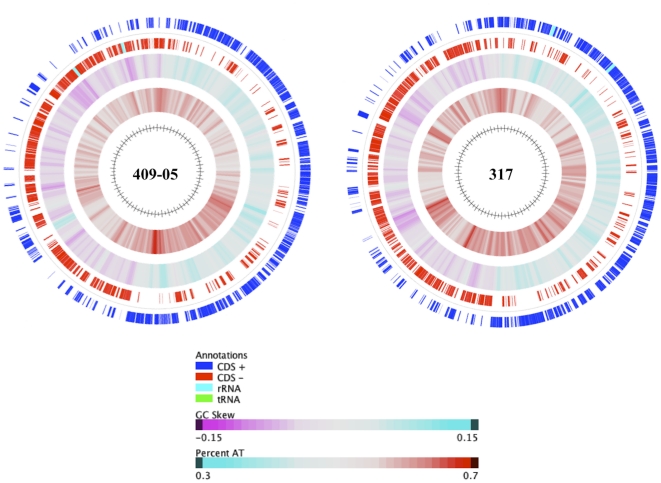
Genome atlases. Genome atlases of the two *G. vaginalis* strains with completed genome sequences: 409-05 (left) and 317 (right). From outside to inside the circles illustrate ORFs of the ‘+’ (1) and ‘−’ (2) strands, GC Skew (3) and % AT (4).

**Figure 3:**
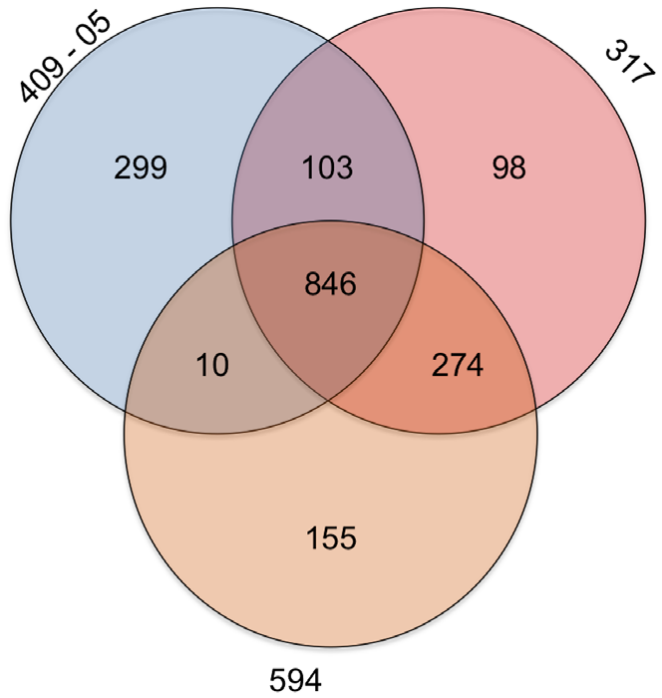
Orthologue distribution. Venn diagram showing the number of orthologues shared between the three strains of *G. vaginalis*.

The general features of the three *G. vaginalis* strains are shown in Table 1. The genome of *G. vaginalis* strain 409-05 (1,617,545 bp) is smaller than that of strain 317 (1,667,350 bp), both being consistent with previous estimates by Lim *et al.* [14]. Their genomes are smaller than other sequenced members of the *Bifidobacteriaceae* family, which range from 1.9–2.8 Mb. All three of the *G. vaginalis* strains appear to have a single chromosomal genomic architecture, with no evidence of episomal elements. The two closed genome sequences (from strains 409-05 and 317) have a standard GC skew, suggesting replication occurs through a typical bidirectional theta mechanism (Fig. 2). All three genomes have a low %GC content (41–42%) although there appeared to be some variation throughout each and in particular the genome of strain 317 (Fig. 2). Strain 317 has 63 more protein encoding genes than strain 409-05, which is solely accounted for by the larger genome. Their comparison provides early evidence of a large pangenome for *G. vaginalis*, with the strains sharing a core genome of just 846 orthologues and 939 strain-variable genes (949 and 681 genes, respectively, if just considering the two complete genome sequences; Fig. 3). Consistent with the 16*S* rDNA phylogenetic similarity, the two *G. vaginalis* strains isolated from BV patients share more orthologues (n = 1120; Fig. 3), and just eight of those orthologues had less than 97.5% amino acid sequence identity (Fig. S1 and Tables 2–3). In contrast almost half (46% and 47%, respectively) of the orthologues shared by strain 409-05 and either strain 317 and/or 594 have less than 80% amino acid sequence identity (Fig. S1).

**Table 1:** General genome features.

Strain	409-05	317	594^1^
**Available non-redundant genome sequence (bp)**	1,617,545	1,667,350	1,603,713
**Genome status**	Closed	Closed	Draft (5x)
**Contigs**	1	1	145
**% G+C content**	42	41	41
**Coding density (%)**	86	86	82
**Genes**	1,258	1,321	1,285
**Average gene length (bp)**	1,091	1,077	1,016
** Assigned function**	918	1103	935
**Enzymes**	450	487	465
**Transport systems**	55	65	65
**ABC-type** 2	25	41	34
**MFS**	6	6	6
**PTS**	1	1	2
**Hypothetical**	185	153	179
**Conserved hypothetical**	141	208	132
**Conserved domain**	88	13	39
**Lipoproteins**	6	4	5
**RNAs**			
**rRNA operons**	2	2	2
**tRNAs**	45	45	34
**ncRNAs**	3	3	4

^**1**^Sequence, and consequently statistics, are incomplete.

2 ABC transporters were defined to consist of, at least, a permease and either a cognate ATP-binding protein or a substrate binding protein.

**Table 2:** TA system components and other competitive exclusion genes.

409-05 (a)	317 (b)	594 (c)	Product	Orthology (% ID) a-b/b-c/c-a
HMPREF0424_0078	n/a	n/a	HicB-family TA system antitoxin	-/-/-
HMPREF0424_0151	n/a	n/a	Fic-family TA system toxin	-/-/-
HMPREF0424_0152	HMPREF0421_21368	158	Fic-family TA system antitoxin	92/100/92
HMPREF0424_0173	n/a	n/a	TA-system toxin	-/-/-
HMPREF0424_0174	HMPREF0421_21334	1274	TA-system antitoxin	98/100/98
HMPREF0424_0193	n/a	n/a	RelE-family TA system toxin	-/-/-
HMPREF0424_0194	n/a	n/a	RelE-family TA system antitoxin	-/-/-
HMPREF0424_0434	n/a	n/a	TA system toxin	-/-/-
HMPREF0424_0435	n/a	n/a	TA system antitoxin	-/-/-
HMPREF0424_0507	HMPREF0421_21336	1275	RelE-family TA system toxin	96/100/96
HMPREF0424_0508	n/a	n/a	RelE-family TA system antitoxin	-/-/-
HMPREF0424_0512	HMPREF0421_21058	608	HipB-family TA system antitoxin	98/100/98
HMPREF0424_0512	HMPREF0421_21057	609	PHD-family TA system antitoxin	99/100/99
HMPREF0424_0517	HMPREF0421_21055	610	HigA-family TA system antitoxin	99/100/99
HMPREF0424_0518	n/a	n/a	RelB-family TA system antitoxin	-/-/-
n/a	HMPREF0421_21053	612	RelE-family TA system toxin	-/100/-
n/a	HMPREF0421_21052	613	RelE-family TA system toxin	-/100/-
HMPREF0424_0564	HMPREF0421_21006	982	HicB-family TA system antitoxin	93/100/93
HMPREF0424_0746	n/a	n/a	RelB-family TA system toxin	-/-/-
HMPREF0424_0747	HMPREF0421_21051	614	RelB-family TA system antitoxin	88/100/88
HMPREF0424_1061	n/a	n/a	RelB-family TA system antitoxin	-/-/-
HMPREF0424_1165	n/a	n/a	HipB-family TA system antitoxin	-/-/-
HMPREF0424_1226	n/a	n/a	TA system antitoxin	-/-/-
n/a	HMPREF0421_20168	929	Fic-family TA system toxin	-/100/-
n/a	HMPREF0421_20084	106	Abortive infection protein	-/100/-
**Other genes with potential roles in competitive exclusion**				
HMPREF0424_0416	n/a	n/a	Abi-like protein	-/-/-
HMPREF0424_0432	n/a	n/a	Abi-like protein	-/-/-
HMPREF0424_0398	HMPREF0421_20542	682	CHAP domain protein	49/100/49
HMPREF0424_1070	HMPREF0421_20929	51	CHAP domain protein	72/100/71
HMPREF0424_1002	n/a	n/a	Lysozyme, LysA	-/-/-
HMPREF0424_1190	n/a	n/a	GH25 enzyme, LysB	-/-/-
HMPREF0424_0641	HMPREF0421_20698	19	SalY-family antimicrobial peptide ABC transport system, ATP-binding protein	96/100/96
HMPREF0424_0642	HMPREF0421_20699	20	SalY-family antimicrobial peptide ABC transport system, permease	93/100/93

n/a - indicates protein was not identified within the genome.

a 409-05.

b 317.

c 594.

**Table 3:** Genes potentially important to biofilm formation and epithelial adhesion.

409-05 (a)	317 (b)	594 (c)	Product	Orthology (% ID) a-b/b-c/c-a
**EPS production and Biofilm formation**				
HMPREF0424_0402	HMPREF0421_20545	685	Family 1 glycosyltransferase	90/100/88
HMPREF0424_1181	n/a	n/a	Family 1 glycosyltransferase	-/-/-
HMPREF0424_0821	HMPREF0421_20842	565	Family 2 glycosyltransferase	81/100/81
HMPREF0424_1138	HMPREF0421_20434	184	Family 2 glycosyltransferase	92/100/92
HMPREF0424_1180	n/a	n/a	Family 2 glycosyltransferase	-/-/-
HMPREF0424_1189	n/a	n/a	Family 2 glycosyltransferase	-/-/-
n/a	HMPREF0421_20405	535	Family 2 glycosyltransferase	-/100/-
n/a	HMPREF0421_20407	537	Family 2 glycosyltransferase	-/100/-
n/a	HMPREF0421_20412	540	Family 2 glycosyltransferase	-/100/-
n/a	HMPREF0421_20413	541/1237	Family 2 glycosyltransferase	-/100/-
HMPREF0424_1083	HMPREF0421_20944	262	Family 4 glycosyltransferase	96/100/96
HMPREF0424_0590	HMPREF0421_20996	224	Glycosyltransferase (unclear family)	57/100/61
**Fimbria/Pili biogenesis (Epithelial adhesion)**				
HMPREF0424_1026	HMPREF0421_21115	749	Type-I fimbrial major subunit precursor	61/100/61
n/a	HMPREF0421_20500	n/a	Type-I fimbrial major subunit precursor	-/-/-
HMPREF0424_1164	HMPREF0421_21204	1121	Type-II fimbrial major subunit precursor	43/100/44
HMPREF0424_0378	HMPREF0421_21089	774	Type IV prepillin peptidase	52/100/52
HMPREF0424_0125	n/a	n/a	TadE-like protein	-/-/-

n/a - indicates protein was not identified within the available genome sequence.

a 409-05.

b 317.

c 594.

All strains have two ribosomal RNA (rRNA) operons, consistent with a previous analysis of strains 317, 594 and several other tested *G. vaginalis* strains. Strains 409-05 and 317 share the same number of transfer RNAs (tRNAs; n = 45), while 34 are currently evident in the strain 594 genome. None of the tRNAs found in the three strains were unique in terms of amino acids transferred or recognized anti-codons (Table S1).

### Genome synteny

**Figure 4:**
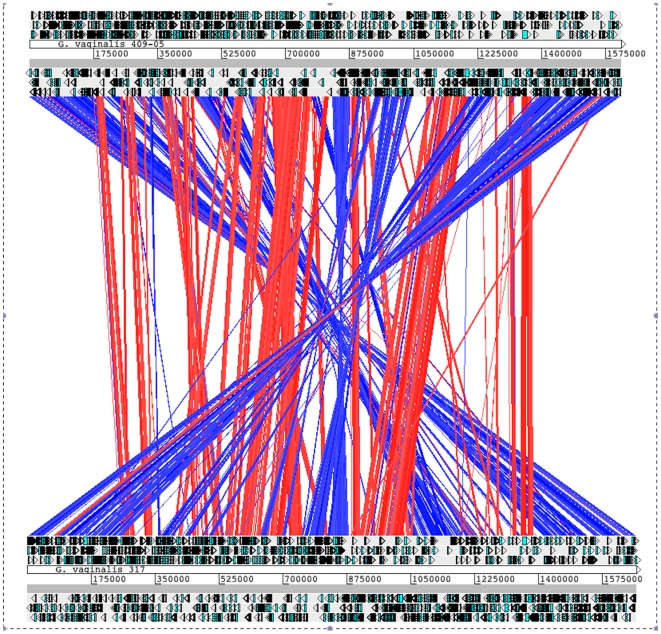
Genome Synteny. Overall synteny between *G. vaginalis* strain 317 (top) and strain 409-05 (bottom). Best Blastn alignments are indicated by a red (same strand) or blue (opposite strand) line and indicate 40–100% ID (illustrated by the light to dark nature of the lines, respectively) over a minimum of 125 contiguous bp. ORFs of the ‘+’ (above) and ‘−’ (below) strands are indicated surrounding the strain and contig information.

Whole genome comparisons of strains 409-05 and 317, the two *Gardnerella* strains with closed genomes show substantial genomic rearrangement, including a large ca. 500 kb inversion encompassing 413 genes, including the region encoding the chromosomal replication initiation protein, DnaA, the rRNA operons, as well as the replication origin, which is typically proximal to *dnaA* (Fig. 4). Evidence presented below suggests both mobile DNA elements and lateral gene acquisition through inducible competence may have played a role in the rearrangement and heterogeneity observed in these genomes.

### Competence and indications of lateral gene transfer

All three strains were found to encode the competence-promoting proteins ComEA, ComEC and CinA (Table S2). Orthologues of ComEA, ComEC and CinA have been identified in many Gram-positive (and Gram-negative) bacteria found to be naturally competent, including *Bacillus subtilis* [15], *Streptococcus pneumoniae* [16] *Thermoanaerobacter spp*. [17] and *Thermus thermophilus* [18]. ComEA and ComEC, have been shown to be involved in the transport of single-stranded DNA across the membrane, where it is then bound by CinA-localized RecA (also present in all three genomes) and integrated into the genome [19]. In characterized systems knockouts of any one of these genes results in the dramatic reduction or inhibition of transformability [15], [17].

To gain further insight into the potential contributions of lateral gene transfer (LGT) in shaping the *G. vaginalis* genomes, we initially determined the propensity of the genes encoded by each of the *G. vaginalis* strains to match the respective codon usage mode of that genome. As genes that differ from this ‘native’ codon usage are potential candidates for LGT [20]. Analyses revealed that a significant number of genes (n = 832, 892 and 889 in strains 409-05, 317 and 594, respectively) did not match the modal codon usage of their respective genome (p<0.1). Ninety, 93 and 75 of these, respectively, were genes that typically have a high basal level of expression and were expected to differ from the mode due to their optimized codon frequencies. Constructing an axis from the mode of the overall genome to the mode of the highly expressed genes, as previously described [20], we found, in total, just 597, 598 and 570 genes, respectively, that based upon their codon adaptation were native to the *G. vaginalis* genomes (p>0.1). Indicating the remaining 53–56% (661, 723 and 715 genes, respectively), were potentially foreign to these genomes.

Work by Davis and Olsen [20], analyzed 923 genomes available in the SEED database [21] using their Native codon usage analysis software; we placed the three *G. vaginalis* strains within this data set (Jim Davis, personal communication) and found each strain to be within the 98^th^ percentile for the fraction of genes not matching the native codon axis (Fig. S2) along with *Neisseria spp*, which are known to be naturally competent [22]. These findings support the hypothesis that LGT has contributed to the genetic composition of each of the *G. vaginalis* genomes. Consistent with this hypothesis 290, 253 and 280 genes, respectively, did not have orthologues in the completed genomes of *Bifidobacterium adolescentis* ATCC15703, *B. dentium* BD1, *B. longum* NCC2705 or in the draft genomes of *B. breve* DSM 20213 and *B. catenulatum* DSM16992 suggesting these genes were either lost in these *Bifidobacterial* strains, had diverged significantly or had been acquired by *G. vaginalis* following their phylogenetic split in the *Bifidobacteriaceae* (refer to Fig. 1 and listed in Table S3). BlastP alignments of each of these genes against the NCBI non-redundant database revealed the best non-*Gardnerella* hits (e-value <0.1; BBH) to be mostly to human associated isolates particularly from the genera *Lactobacillus* (47 genes), *Ruminococcus* (12 genes), *Rothia* (9 genes), *Coprococcus* (8 genes), and *Oribacterium* (6 genes) and included common vaginal isolates *Atopobium vaginae* (57 genes), *Lactobacillus iners* (27 genes), *L. jensenii* (3 genes), *Mobiluncas mulieris* (12 genes) *Peptostreptococcus anaerobius* (4 genes), *Actinomyces urogenitalis* (3 genes), *Anaerococcus lactolyticus* (2 genes) and *Streptococcus mitis* (2 genes). Several genes also aligned to more distantly-related members of the *Bifidobacteriaceae*, including those from the genera *Scardovia* (10 genes) and *Parascardovia* (15 genes) supporting the former hypothesis that some of these genes had been lost in more closely-related *Bifidobacteria*.

The majority of these genes (67%) were unable to be assigned an annotated function but additionally included many of the genes identified as being potentially important to virulence (discussed below) including vaginolysin (BBH: *Streptococcus intermedius*), isochorismatase (BBH: *Atopobium vaginae*), the Rib-family protein (BBH: *Lactobacillus iners*), the GA module protein (BBH: *Lactobacillus jensenii*), several antibiotic resistance proteins including those potentially important to methicillin (BBH: *Mobiluncus mulieris*) and lantibiotic (BBH: *Clostridiales genomosp.*) resistances, as well as many genes potentially important to biofilm formation, adhesion to the epithelium and nine TA system toxin or antitoxin components. In addition to natural competence, other potential mechanisms for LGT exist and in *G. vaginalis* may include transposon or phage-mediated gene shuttling through mobile elements.

### Mobile elements

Accounting for the large 500-kb inversion observed between strains 409-05 and 317, the first loss of gene synteny between these strains corresponds to the insertion of an IS*3509*a-family transposon in strain 317. The IS*3509*a-family compound transposon comprises 21 genes (HMPREF0421_20010–HMPREF0421_20030), flanked by an annotated IS*3509*a-family transposase and a recombinase. Genes annotated to encode a transcriptional regulator (HMPREF0421_20023), a toxin-antitoxin (TA) system toxin (HMPREF0421_20022) and two permeases map within the transposon; the remaining genes were unable to be assigned a function. The permeases appear to be specific for the efflux of multiple drugs (HMPREF0421_20015) and the uptake of colicin (HMPREF0421_20011), respectively. Orthologues of nine of the twenty one IS*3509*a-family transposon genes, including the transposase and recombinase (open reading frames (ORFs) 433 and 1192, respectively), were identified in strain 594, however none of the twenty one genes were found in the genome of strain 409-05, suggesting the IS3509a-family transposons acquisition was a recent event on an evolutionary timescale. Strain 409-05 contains just a single transposase belonging to IS*150* family (HMPREF0424_509; Table S4). Each strain possessed several bacteriophage-associated genes though most of those present in either strain 317 or 594 did not have orthologues in strain 409-05 and vice versa.

### Toxin-Antitoxin system mediated competitive exclusion systems

Despite the paucity of transposases, a notable feature of each *G. vaginalis* genome is an abundance of TA systems. TA systems typically comprise two genes; one that encodes a stable toxin that would be harmful to the host cell if it was not for the expression of a more labile cognate antitoxin. They are self-promoting as the loss of either the antitoxin component or both causes the cell to lose viability, and are often found on mobile elements [23]. Strain 409-05 encodes six TA system toxins, while strains 317 and 594 each encode four. The toxins represent an assortment of TA system families (Table 2). While these are likely remnants from prior infection of mobile elements, their retention may provide *G. vaginalis* with a competitive advantage over other vaginal colonists through the production of an assortment of toxins.

Recently, it has been shown TA system toxins can be delivered to co-inhabitants via a type VI secretion system and effectively retard their growth [24]. For strain 409-05, all of the toxin genes are contiguous with those encoding annotated cognate antitoxin components, while three of the toxins in strains 317 and 594 show no evidence of a contiguous antitoxin component. Each strain encodes many more antitoxins than toxins. Strain 409-05 encodes 15 antitoxin components, while strains 317 and 594 each encode eight. The unpaired antitoxin genes may be retained to enable resistance to toxins produced by other bacterial strains that are co-inhabitants of the vaginal biome. Consistent with the hypothesis that these TA systems are being retained for competitive purposes, the components are strongly conserved between strains and six (five in the BV-isolates) of the TA system genes are clustered within the *G. vaginalis* genomes. Collectively, these findings suggest that *G. vaginalis* may competitively exclude a wide-range of vaginal co-inhabitants and is resistant to a larger number of bacterial toxins than either of the strains produce.

### Competitive exclusion

In addition to the TA systems, each strain possesses additional genes likely to promote competitive exclusion. All three strains encode two CHAP domain proteins and an ABC-transport system involved in the secretion of antimicrobials (Table 2). A recent study of the CHAP domain found it to have a strong lytic ability [25]. Strain 409-05 uniquely encodes two glycoside hydrolase (GH) family 25 proteins, a family that exclusively comprises lysozyme, and two Abi-proteins. Recent analysis of Abi-proteins has shown they confer resistance to a broad-range of related bacteriocins [26]. These findings are in agreement with a recent publication that found *G. vaginalis* strains produced substances that were antagonistic to bacterial isolates common to the vaginal biome [27].

### Metabolism

**Figure 5:**
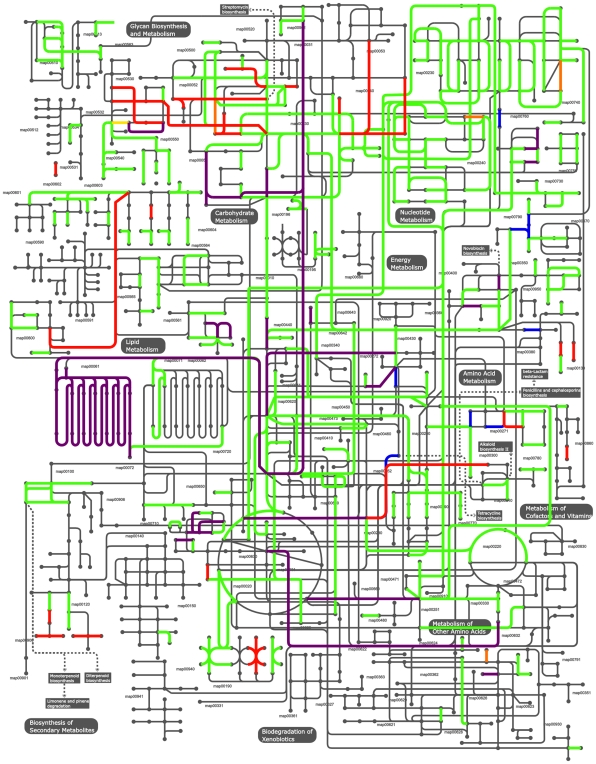
Metabolic potential. The metabolic pathways of *G. vaginalis* were mapped based upon genome information using iPath. Common pathways are shown (green), along with those specific to strain 409-05 (blue), 317 (orange) and 594 (yellow). Those pathways common to the two BV-isolates are shown in red, while those common to strain 409-05 and 317 are shown in purple. No enzymes were exclusively found in 409-05 and 594.

Based on the closed genome sequences, the predicted metabolic capabilities of strains 409-05 and 317 were modeled and compared (Fig. 5). This analyses focuses on those with predicted enzymatic activity and ignores other proteins, including those involved in substrate transport, which are abundant in all strains (Table 1). Approximately one third of the genes identified in strains 409-05 (n = 450) and 317 (n = 487) (currently strain 594 has 465) were determined to encode enzymes. The majority of these enzymatic functions were shared by all three genomes (n = 429). In terms of lifestyle, all strains appear capable of catabolizing glycogen and glucose, the most abundant carbohydrate sources in the vagina [28], along with other less abundant carbohydrate sources such as fructose and starch. All strains were equipped with numerous (n = 13–14) proteases and peptidases that may be involved in proteolysis for nitrogen (excluding signal peptidases, pre-pilin peptidases, penicillin-binding protein transpeptidases and those involved in cellular homeostasis, e.g. FtsH). Each strain is predicted to be capable of using amino acids and ammonia as nitrogen sources, but no strain appeared capable of using urea (Table S5). Major metabolic pathways including glycolysis and the pentose-phosphate pathway are present, however in contrast to most *Bifidobacteria*, both *G. vaginalis* strains appear to have lost most of the TCA cycle, retaining only succinate dehydrogenase (EC 1.3.99.1) and malate dehydrogenase (EC 1.1.1.37; Table S7).

A number of differences were observed between the strains (Fig. 5 and Table S6) in various metabolic pathways including carbohydrate, amino acid, nucleic acid and vitamin metabolisms. As genes present in strains 409-05, 317 or both, but not in 594 may be present in the missing genome sequence of strain 594, those differences will not be discussed in text. Of particular interest were the differences observed in either or both of the two strains isolated from patients with symptomatic BV, but not seen in strain 409-05. These included pathways enabling the catabolism of both galactose and arabinose (Table S7), and enzymes involved in terpenoid biosynthesis.

A particularly significant difference among the strains was the encoded ability of the two BV-isolates to degrade the N- and O-glycan portions of glycoproteins (Table 4). Glycoproteins are the primary component of mucins that are secreted to form the protective barriers of host mucosal epithelial layers. In contrast, strain 409-05, isolated from an asymptomatic individual, does not have this capacity. The presence of genes in the two BV-isolates involved in the catabolic degradation of N-acetylglucosamine (the major sugar in glycans) further emphasizes this difference and suggests that these strains may have a greater propensity to invade the mucosa and that this may be important to the causation of symptomatic BV. Previous measures of mucolytic enzymes in the vaginal cavity show strong correlations with BV [29], [30]. The ability to degrade mucins has been proposed to be a necessary step in the colonization of vaginal epithelial cells and in the displacement of the normally dominant *Lactobacilli* [31]. Further, there is evidence that the degradation of vaginal mucins impairs specific-immunoglobulin A immune responses [29]. Given this, along with the importance of glycoproteins to the integrity, growth and function of vaginal epithelial cells, disruption of the mucin-layer may predispose hosts to further complications, such as those currently associated with BV, including an increased risk of infection with HIV and other sexually transmitted diseases [8]. All three strains encode glycoproteases (EC 3.4.24.57) suggesting each can exploit glycoproteins as a nitrogen source, though the BV-isolates are able to exploit the carbohydrate component. None of the strains were annotated as encoding enzymes enabling the utilization of fucose or mannose.

**Table 4:** Potential virulence genes.

409-05 (a)	317 (b)	594 (c)	Product	Orthology (% ID) a-b/b-c/c-a
**Cytotoxicity**				
HMPREF0424_0103	HMPREF0421_20066	992	Vaginolysin	94/100/94
HMPREF0424_0679	HMPREF0421_20593	655	rRNA methyltransferase (possible TlyA-family hemolysin)	75/100/75
**Antibiotic/Antimicrobial resistance**				
HMPREF0424_0074	HMPREF0421_20093	98	MATE-family multidrug efflux permease	71/100/71
HMPREF0424_0156- HMPREF0424_0158	HMPREF0421_21363- HMPREF0421_21361	163–165	Lantibiotic resistance ABC transporter	≥98/100/≥98
n/a	HMPREF0421_20446	954	Methicillin resistance protein	-/100/-
HMPREF0424_0354	HMPREF0421_20418	1243	Multidrug resistance antiporter	83/100/83
HMPREF0424_1122	HMPREF0421_20428	192	Multidrug resistance ABC transporter	97/100/95
HMPREF0424_1123	HMPREF0421_20427	191	Multidrug resistance ABC transporter	94/100/94
HMPREF0424_0217	HMPREF0421_20340	283	Bleomycin hydrolase	91/100/91
n/a	HMPREF0421_20507	1079	Aminoglycoside phosphotransferase	-/100/-
HMPREF0424_0210	HMPREF0421_20333	276	DedA-family protein	92/100/92
**Iron acquisition**				
HMPREF0424_0013	HMPREF0421_20160	1120	Ferritin	92/100/87
HMPREF0424_0160	HMPREF0421_21358	168	FTR1-family iron permease	80/100/79
HMPREF0424_0161	HMPREF0421_21357	169	TPD-family pathogen-specific lactoferrin binding protein	94/100/88
n/a	HMPREF0421_20888	n/a	Oxygen-independent coproporphyrinogen III oxidase	-/-/-
HMPREF0424_0852	HMPREF0421_20889	823	Oxygen-independent coproporphyrinogen III oxidase	94/100/88
HMPREF0424_1040	HMPREF0421_20881	138	FUR-family transcriptional regulator	83/100/83
HMPREF0424_1242	HMPREF0421_20302	1223	Isochorismatase family protein	97/100/97
**Mucin degradation**				
n/a	HMPREF0421_20186	n/a	Sialidase A precursor	-/-/-
n/a	HMPREF0421_20101	1135	α-L-fucosidase	-/100/-
n/a	HMPREF0421_20100	1134	β-galactosidase	-/100/-
HMPREF0424_1049	HMPREF0421_20907	1043	α-Mannosidase	92/100/93
HMPREF0424_0937	HMPREF0421_20740	1141	O-Sialoglycoprotein endopeptidase	95/100/95
HMPREF0424_0939	HMPREF0421_20738	1139	M22-family glycoprotease	66/100/66
**Protection from or evasion of immune response**				
HMPREF0424_0003	HMPREF0421_20840	567	Peroxiredoxin	91/91/100
HMPREF0424_1196	HMPREF0421_21226	n/a	Rib-family surface protein	76/-/-
**Other virulence-related genes**				
HMPREF0424_0399	HMPREF0421_20543	683	Invasion-associated hydrolase	70/100/65
HMPREF0424_0545	HMPREF0421_20447	427	GA module protein	50/100/50
HMPREF0424_0888	HMPREF0421_20630	972	NLPA lipoprotein	85/100/85
HMPREF0424_1075	HMPREF0421_20934	n/a	Endothelin-converting enzyme	91/-/-
HMPREF0424_1186	n/a	n/a	LicD protein	-/-/-
HMPREF0424_1263	HMPREF0421_20273	114	Raf-like phospholipid-binding protein	77/100/77
n/a	n/a	237	Oxygen-insensitive NADPH nitroreductase, RdxA	-/-/-

n/a - indicates protein was not identified within the available genome sequence.

a 409-05.

b 317.

c 594.

### Virulence

Several common features of virulence are evident in the genomes of one or more of the *G. vaginalis* strains including mucin degradation (described above), cytotoxicity, hemolysis, adhesion to the epithelium, biofilm production, iron scavenging, and antimicrobial resistance (Tables 3 and 4).

#### Effector protein translocation

A few genes implicated in effector protein translocation were identified in all three strains (Table S8). These genes appear to be randomly distributed throughout each genome suggesting such a system may exist. However, as secretion systems are less well elucidated in Gram-positive organisms as compared to their Gram-negative counterparts, the presence or absence of a secretion system is not conclusive.

#### Epithelial adhesion

The ability of *G. vaginalis* to adhere to vaginal epithelial cells has been demonstrated [6]. Epithelial adhesion is typically mediated by pili [32]. Genes encoding both type I and II pili are present in each strain (Table 3). The presence of a type IV prepilin peptidase suggests that they may also encode a type IV Flp pilin/pseudopilin. Although there is no Flp1 pilin gene apparent in the *G. vaginalis* genomes, strain 409-05 uniquely encodes TadE. The *tadE* gene typically occurs as part of a larger type IV pilin-related gene cluster, and the protein resembles the domain architecture of some Flp1 pilins. Like the Flp1 pilins, TadE is post-translationally processed by prepilin peptidases, but are considered pseudopilins [33]. In *Aggregatibacter actinomycetemcomitans* (formerly designated *Actinobacillus actinomycetemcomitans*), TadE has been shown to be important to epithelial adhesion [33]. The same study found preprocessed TadE to be important for biofilm formation.

#### Biofilm production

*G. vaginalis* can form a biofilm [6]. Our genomic analysis suggests this is, at-least in part, due to predominantly type II, but also types I and IV glycosyltransferases (GTs). Strain 409-05 uniquely encodes eight GTs, while the two BV-isolates (317 and 594) encode nine GTs that are likely to be important for the biosynthesis of exopolysaccharide (EPS) for biofilm formation (Table 3). Five of the GTs appear to be orthologous among all strains, while three 409-05 GTs showed little or no sequence similarity to genes of the other two strains. All GTs present in strain 317 were identified in the available genome sequence of strain 594 and had 100% amino acid identity. Biofilm formation also typically involves additional factors, in addition to those facilitating EPS formation. In Gram-positive bacteria this commonly includes proteins carrying an LPxTG motif that are attached to the cell surface by a sortase enzyme. Each of strain 409-05, 317 and 594 are predicted to encode 6, 4 and 4 sortase enzymes, respectively, and 15, 13 and 12 proteins, respectively, carrying an LPxTG motif (Table S9). These include a Rib protein (HMPREF0424_1196, HMPREF0421_21226) and a protein with two G-related albumin-binding (GA) modules (HMPREF0424_0399, HMPREF0421_20447 and ORF 683), both which have further virulence potential (as discussed below), along with two of the proteins predicted to be involved in the biogenesis of fimbria/pili (HMPREF0424_1026 and HMPREF_0424_1164), each of which may additionally contribute to biofilm formation.

#### Cytotoxicity/hemolysis

The hemolytic activity of *G. vaginalis* was first described in 1955 [12] and has since been attributed to a single protein that is excreted from *G. vaginalis* cells during exponential growth [34]. The lytic activity of the toxin is specific for human erythrocytes, neutrophils and endothelial cells [34], [35]. The 59-kDa hemolysin was first isolated and characterized by Cauci *et al.* [29]. The thiol-activated cytolysin of *G. vaginalis* strain 594 was subsequently cloned, sequenced (NCBI accession ACD39461) and characterized by Gelber *et al.* [35] and found it to be a cholesterol-dependent cytolysin (CDC) family toxin, which the authors designated vaginolysin. Vaginolysin (HMPREF0424_0103, HMPREF0421_20066 and ORF 992 in strains 409-05, 317 and 594, respectively) is encoded by all three *G. vaginalis* strains, is highly conserved between strains 409-05 and 317 (94% aa ID) and is identical in strains 594 and 317. The predicted molecular masses (56.7 kDa for 409-05 and 60 kDa for 317 and 594) along with predicted extracellular localizations of vaginolysin in each strain is consistent with previous observations. Vaginolysin is also strongly conserved in *G. vaginalis* strain T11 (95% amino acid identity; NCBI accession number ACD63042 [36]), from which the encoding genes have been sequenced. Another frequent vaginal inhabitant, *Lactobacillus iners*, also possesses a vaginolysin homolog, annotated as perfringolysin O in the *L. iners* strain DSM 13335 (NCBI accession number ZP_05744302, 94% amino acid identity, e-value 2×10^−148^). In addition to vaginolysin, we found evidence for the existence of a second hemolytic/cytolytic toxin in each strain (HMPREF00424_0679, HMPREF0421_20593 and 655, for strains 409-05, 317 and 594 respectively; Table 4). The collective evidence used to annotate this protein suggested it was best annotated as an rRNA methyltransferase (see Text S1), but the amino acid sequence aligns strongly (e-values  = 2×10^−36^–4×10^−42^) to the TIGRFAM TIGR00478, for which two characterized members were found to be hemolytic (see Text S1).

#### Iron acquisition

In addition to hemolytic activity, each of the *G. vaginalis* strains appears capable of acquiring iron through at least two high-affinity iron transporters, consistent with previous reports [37]. These include an FTR1-family high-affinity iron transporter and a Tpd-family pathogen-specific lactoferrin-binding protein thought to function as a second high-affinity iron transporter (Table 4). In a previous study by Jarosik *et al.* [37], strains 594 and 317 along with seven other *G. vaginalis* strains were found to produce siderophores. In trying to determine the type of siderophore produced and enzymatic machinery responsible, we identified isochorismatase (E.C 3.3.2.1; Table 4), an enzyme involved in the production of the siderophores vibriobactin, enterochelin and bacillibactin (KEGG pathway KO1053). Other enzymes important to siderophore biosynthesis proved more elusive, though two proteins annotated as 3-oxoacyl-[acyl-carrier-protein] reductases in both the *G. vaginalis* 594 and 317 genomes (HMPREF0421_21015 and HMPREF0421_21034 in strain 317 and ORFs 1261 and 880 in strain 594) resemble the 2,3-dihydro-2,3-dihydroxybenzoate dehydrogenase (EC 1.3.1.28) of *Stenotrophomonas maltophilia* (e-values 2×10^−10^–6×10^−17^; NC_010943.1), an enzyme involved in the same siderophore biosynthetic pathway. The genes HMPREF0421_21015 and 1261 also matched strongly to the Hidden Markov Model PRK08220, that describes 2,3-dihydro-2,3-dihydroxybenzoate dehydrogenases (e-value  = 2×10^−47^). No orthologue of this enzyme, or other potential candidate for a 2,3-dihydro-2,3-dihydroxybenzoate dehydrogenase was identified for strain 409-05.

Strain 409-05 also encodes an oxygen-independent coproporphyrinogen III oxidase (HemN), while strain 317 and 594 encode two. HemN is involved in the breakdown of porphyrins, such as in hemoglobin or myoglobin, to release iron. Each strain encodes a Ferric uptake regulator (FUR)-family global iron-binding transcriptional repressor. As Fur-family repressors often regulate genes involved in iron utilization [38], we attempted to glean more information by identifying the DNA elements that bind Fur-family regulators, namely Fur boxes (see methods). Several FUR-box candidates were identified (Text S2 and Table S10), though most occurred in agenic regions, although one Fur-box was identified downstream of the isochorismatase in both strains 409-05 and 594, but not in strain 317.

#### Antibiotic/antimicrobial resistance

Genes encoding antimicrobial-specific resistance proteins that were identified in each strain included those conferring resistance to bleomycin and an unknown lantibiotic. Strains 317 and 594 also encoded genes promoting resistance to methicillin and aminoglycosides (Table 4). In *Staphylococci*, FemAB-family proteins (orthologous to HMPREF0421_20446 and ORF 954) are involved in the formation of pentaglycine interpeptide bridges, but have also been found to be essential to methicillin resistance [39], [40]. Although methicillin was not introduced until after strains 317 and 594 were isolated [41], the precursors of a resistance mechanism were available. In contrast, aminoglycosides like streptomycin and neomycin were used to treat vaginal infections at the time strains 317 and 594 were isolated [42]. These strains each encode an aminoglycoside phosphotransferase (HMPREF0421_20507 and ORF 1079), related to that of the more modern nosocomial pathogen *Stenotrophomonas maltophilia* (e-value 2×10^−27^) [43]. The aminoglycoside phosphotransferase of *S. maltophilia*, and a number of other organisms, has been shown to significantly increase resistance to aminoglycosides and work by inactivating the antibiotics through phosphorylation [41]. Each strain encoded three multi-drug extrusion transporters, and strains 317 and 594 encode a heavy metal exporter that appears to be specific for cadmium (HMPREF0421_21068 and ORF 602, respectively). Collectively, these efflux transporters potentiate a much broader antimicrobial tolerance. It is also likely, given the heterogeneity observed among the *G. vaginalis* strains, that as a species, a much broader antimicrobial resistance complement exists. Like many Gram-positive pathogens, multiple antimicrobial resistances are fast becoming a major problem for the healthcare sector [44], [45]. The potential competence-promoting nature of *G. vaginalis* strains (Table S2 and discussed above) also suggests they may have the capacity to rapidly adapt to new environmental challenges.

#### Immune response evasion and mitigation

Immune responses specific to *G. vaginalis* may be impaired by the ability of the organism to evade detection by altering its surface antigens. Both *G. vaginalis* strains 409-05 and 317, but not 594 encode a Rib-protein. Rib proteins belong to the α-like protein (Alp)-family of highly repetitive surface antigens found in Gram-positive pathogens [46]. These proteins elicit protective immunity through their inter-strain size variability. Size predictions based on the primary amino acid sequences indicate the Rib protein encoded by strain 409-05 is more than 2.5 times larger (predicted to be 338 kDa) than that encoded by strain 317 (predicted to be 128 kDa). Each strain may also be capable of further protecting themselves from macrophage peroxynitrite attack through encoded antioxidant peroxiredoxins (HMPREF0424_0003, HMPREF0421_20840 and ORF 567, respectively), as has been shown in other pathogenic bacteria [47].

#### Other potential virulence factors

Several other genes were identified that have been linked to virulence or pathogenic potential (Table 4). This includes a gene resembling a major virulence factor of *Listeria monocytogenes*. Each strain encodes an invasion-associated hydrolase that has homology with the *L. monocytogenes* p60 (e-value  = 6×10^−17^), an enzyme with murein hydrolase activity that has been linked to fibroblast invasion, immune modulation [48], and complement-independent hepatocyte and macrophage invasion [49]. Other genes of interest include those that encode a novel large (221 kDa) extracellular protein with two GA modules (HMPREF0424_0399, HMPREF0421_20447 and ORF 683) [50] and, in both strains 409-05 and 317, a protein that aligns with the endothelin-converting enzyme (ECE; HMPREF0424_1075 and HMPREF0421_20934; e-value  = 7×10^−77^) from endothelial cells of the Norwegian rat (*Rattus norvegicus*) [51]. In eukaryotic systems ECE activates endothelin-1, a protein responsible for constricting blood vessels and raising blood pressure [52]. The role of ECE within *G. vaginalis* is unclear, but it may help to increase the availability of heme containing red blood cells. Strain 409-05 encodes a LicD-family protein (HMPREF0424_1186). In *Streptococcus pneumoniae*, the LicD protein has been shown to have a role in virulence, specifically in cytoadhesion and mutational inactivation of *licD* has been shown to decrease transformation competence [53]. In the same organism, LicD has also been linked to phosphocholine metabolism [53]. Along with a Raf-like phospholipid binding protein (HMPREF0424_1263, HMPREF0421_20473 and ORF114), which is present in all genomes, LicD may further aid the ability of *G. vaginalis* to adhere to epithelial cells.

### Conclusion

*Gardnerella vaginalis* is one of several vaginal organisms whose presence strongly correlates with BV. Biochemical and physiological analyses demonstrate important virulence features such as hemolytic activity, epithelial adhesion and biofilm formation [6]. Our genomic analyses support these findings and provide detail of the genetic elements involved. We also identify other features of the *G. vaginalis* pangenome, such as the ability to degrade mucin, evade immune detection or resist a broad spectrum of antimicrobials, which may be important to the role of *G. vaginalis* in BV. Overall the two BV-isolates (strains 317 and 594) had a highly similar 16*S* rDNA sequence and genome content. In contrast, substantial differences were observed between the BV strains and 409-05. The BV isolates uniquely encoded proteins enabling the degradation of mucin and had a broader group of antibiotic resistance genes including an aminoglycoside phosphotransferase and the precursors of methicillin resistance. These genes were absent from strain 409-05, which was isolated from an asymptomatic subject with a perturbed vaginal microbiome. Compared to the BV isolates, strain 409-05 encoded more proteins predicted to be involved in competitive exclusion. It will be interesting moving forward to further elucidate the *G. vaginalis* pangenome and determine the commonality of features like mucin degradation, and to determine if such features are enriched in those strains isolated from BV patients. It will also be interesting to determine the capacity of strain 409-05, and others like it, to cause symptomatic BV, or if these strains instead play a role in maintaining a healthy vaginal microbiome through the competitive exclusion of BV-causing isolates. We hope these findings, along with the newly available genome sequences, may enhance the efficacy of research into *G. vaginalis* physiology and provide insights into the contribution of the organism to the microbiome of the host, as well as lead to a more complete understanding of its role in health and disease, in particular its role in BV.

## Methods

### Ethics statement

The isolation of all three *Gardnerella vaginalis* strains has previously been reported, along with appropriate human subject ethics considerations [4], [12]. The work reported here does not describe the isolation of these organisms from any human subject, nor does it directly involve human participants.

### Strain Isolation

*G. vaginalis* strain 409-05 was isolated from the vaginal swab of a healthy female with a high Nugent score of 9 [4], while strains 317 and 594 were isolated from vaginal secretions of BV patients [12]. All strains are available from the ATCC-BEI Global Bioresource center.

### Phylogeny reconstruction

SSU rDNA sequences from related species of the *Bifidobacteriaceae* family were obtained from Greengenes, where available, or from Genbank. Sequences were trimmed to remove overhangs and aligned using ClustalW [54] without gap penalties to preserve structural alignments. As *Rubrobacter xylanophilus* is believed to be one of the deepest branching species of the *Actinobacteria* phyla [55], it was selected for use as the outgroup. Maximum likelihood trees were constructed with 500 bootstraps using RaxML-III [56] and the resulting trees were drawn and analysed using phyloXML [57].

### Genome sequencing

The closed genomes of *G. vaginalis* strains 409-05 and 317 were sequenced as part of the NIH-sponsored Human Microbiome Project (HMP) using 454 pyrosequencing technology and finished by Sanger-sequencing of a 4 Kb library or PCR-based primer-walking, respectively. Strain 409-05 was sequenced to 34× coverage at the J. Craig Venter Institute, while strain 317 was sequenced to 70× coverage at the Baylor College of Medicine. Hybrid assemblies of both Sangar and pyrosequencing data sets were performed using the Celera v5.1 [58] or Newbler v2.3 (454 Life Sciences, CT, USA) assemblers, respectively. The assemblies were manually validated using Consed v19.89 in conjunction with supporting sequence and scaffold information. The draft sequence of strain 594, available in 145 contigs, was produced by the Stanford Genome Technology Center and has 5× coverage. Each sequence has been deposited in GenBank [59] under the accession numbers CP001849, ACGF00000000 and ADNB00000000, respectively.

### Genome analysis

All genes discussed were manually reannotated using information gleaned from the following resources: BlastP [60] searches of both the NCBI non-redundant and SwissProt databases; modular analysis using HMMER 3.0 [61], CDD [62] and InterPro scan [63]; protein localization predictions using Gpos-PLoc [64]; and Protein molecular weight predictions, made using the ExPASy proteomics server (http://ca.expasy.org/tools). Transfer RNAs were determined using tRNAscan-SE [65]. Orthologues shared among the *G. vaginalis* strains were determined using BlastP searches and defined as the best match with >40% identity over >70% of the sequence of the largest protein, with exception of the size-variable Rib protein. A less conservative ID threshold (>30% identity) was used to determine orthologues to bifidobacterial species. Enzyme commission (EC) numbers were assigned using the Kyoto Encyclopedia of Genes and Genomes (KEGG) [66] and metabolic pathways were mapped and analyzed using iPath [67] (http://pathways.embl.de/). All annotations and analyses were undertaken with careful consideration of the surrounding literature and are available in SEED upon request to the authors. Native codon usage analyses were undertaken as described [20], briefly, codon usage modes were determined as previously described [68]. An axis beginning at the modal codon usage of the genome and extending through the point representing the modal codon usage of genes within the genome that are orthologous (e-value ≥1×10^−5^, >70% coverage of the longest gene and >20% sequence ID) to genes that have been shown to be highly expressed in both *Escherichia coli* K-12 and *Methanococcus maripaludis* S2 was then constructed in a 59-dimensional plot where each dimension represents each of the synonymous codons, excluding termination codons. Genes found to match any point along this axis, as determined by the chi-square test (p>0.1; as described in 9) are defined as native to the genome, those not matching the mode are defined as foreign. Genome atlases were created using the GeneWiz browser [69]. The genome synteny plot was created using ACT [70]. For both genome atlas and genome synteny analyses the genome sequences were reordered to begin at the gene encoding DnaA.

## Supporting Information

Text S1 Toxin characteristics. Further discussion surrounding the analyses applied to the toxins of *G. vaginalis*.
(0.07 MB PDF)

Text S2 Identification of Fur-regulated genes. More in-depth discussion surrounding the methodology and analysis of FUR-regulatory elements and their proximal genes.
(0.07 MB PDF)

Figure S1 Orthologue protein sequence identity. Number of determined orthologues sharing each degree of amino acid sequence identity rounded to the nearest 5%.
(0.07 MB PDF)

Figure S2 Proportion of native genes within *Gardnerella vaginalis* relative to other bacteria. The number of genes matching the modal codon usage relative to the number of genes within the genome of *Gardnerella vaginalis* (red) is ploted along with 923 other genomes (blue).
(5.39 MB TIF)

Table S1 Transfer RNA distribution. The number of tRNAs identified in each of the *G. vaginalis* genomes along with the anticodon recognized and amino acid transferred.
(0.10 MB PDF)

Table S2 Genes potentially involved in competence. Genes identified within the *G. vaginalis*genomes whose annotated roles have been shown to promote competence in other bacteria.
(0.06 MB PDF)

Table S3 *G. vaginalis* genes lacking orthologues in Bifidobacteria. These genes had no apparent orthologue in the completed genomes of *Bifidobacterium adolescentis* ATCC15703, *B. dentium* BD1, *B. longum* NCC2705 or in the draft genomes of *B. breve* DSM 20213 and *B. catenulatum* DSM16992. The best non-*Gardnerella* blast matches, locus tags/ORF number and annotated function are also shown.
(0.09 MB XLS)

Table S4 Genes characteristic of mobile elements. Genes identified within each *G. vaginalis* genome that are likely to have been components of mobile elements.
(0.06 MB PDF)

Table S5 Nitrogen metabolism genes. Genes identified within the *G. vaginalis* genomes that appear to encode functions important for the utilization of various nitrogen sources.
(0.08 MB PDF)

Table S6 Strain variable enzymes. Genes identified from one or more of the *G. vaginalis* genomes that are predicted to encode enzymes whose functions do not appear to be encoded for in the other strain(s).
(0.09 MB PDF)

Table S7 Carbohydrate metabolism genes. Genes present in one or more of the *G. vaginalis* genomes that appear to encode functions important to the utilization of various carbohydrates.
(0.09 MB PDF)

Table S8 Genes potentially important to effector protein translocation. Genes identified within each *G. vaginalis* strain that are potentially important to effector protein translocation.
(0.06 MB PDF)

Table S9 Sortase enzymes and proteins cantaining LPxTG motifs. All sortase encoding enzymes and proteins carrying LPxTG motifs within the genomes of the *G. vaginalis* strains. Note LPxTG motif-containing proteins are typically attached to the cell surface by sortase enzymes.
(0.08 MB PDF)

Table S10 Potential FUR-regulatory sequences. All identified FUR-box sequences identified within each *G. vaginalis* strain along with proximal downstream genes, which may be regulated by the FUR-family transcriptional regulator.
(0.08 MB PDF)
