# Supplementary material for: Comparative Genomics of Gardnerella vaginalis Strains Reveals Substantial Differences in Metabolic and Virulence Potential
Source: PLoS One. 2010 Aug 26;5(8):e12411. doi: 10.1371/journal.pone.0012411 (PMC2928729; doi:10.1371/journal.pone.0012411)
Supplement: Text S1 — Toxin characteristics. Further discussion surrounding the analyses applied to the toxins of G. vaginalis. (0.07 MB PDF) [file pone.0012411.s001.pdf]

Text S1 Toxin characteristics

To glean more information about the two potential cytolytic/hemolytic toxins encoded by the *G. vaginalis* strains, we used previously described bioinformatics techniques to predict their molecular mass and localization. Molecular weight predictions, made using the ExPASy proteomics server (http://ca.expasy.org/tools), while protein localization predictions were made using Gpos-PLoc (Shen and Chou 2007) and the SignalP and LipoP HMMs through InterPro scan (Hunter *et al.* 2009). Consistent with previous reports (Rottini *et. al.*, 1990), vaginolysin (HMPREF0424_0103, 57 and 992 in 409-05, 317 and 594, respectively) is predicted to be secreted.

The potential TlyA-family hemolysin (HMPREF0424_0679, 577 and 655, respectively) has more evidence suggesting an RNA methyltransferase activity. This included both FtsJ-like S-adenosylmethionine-dependent methyltransferase (pfam01728) and S4-family RNA-binding (pfam01479) hidden Markov model (HMM) matches. Evidence for potential hemolytic activity included the TIGR00478 HMM, which describes a group of proteins that best resemble methyltransferases with RNA-binding ability, but for which two members have been characterized and found to have hemolytic activity (Muir *et. al.*, 1992; Wren *et. al.*, 1998).
