# Supplementary material for: Comparative Genomics of Gardnerella vaginalis Strains Reveals Substantial Differences in Metabolic and Virulence Potential
Source: PLoS One. 2010 Aug 26;5(8):e12411. doi: 10.1371/journal.pone.0012411 (PMC2928729; doi:10.1371/journal.pone.0012411)
Supplement: Text S2 — Identification of Fur-regulated genes. More in-depth discussion surrounding the methodology and analysis of FUR-regulatory elements and their proximal genes. (0.07 MB PDF) [file pone.0012411.s002.pdf]

Text S2 Identification of Fur-regulated genes

We attempted to further determine the type of siderophore produced and enzymatic pathway(s) involved. As enzymes required for the biosynthesis of siderophores are commonly transcriptionally regulated by Fur-family global iron-binding transcriptional repressors (Escolar *et al.*, 1999) and homologues of this protein was encoded by both genomes, we went about identifying the DNA elements that bind Fur-family regulators, Fur boxes, as has been used previously to successfully (Najimi *et al.*, 2008). Fur-boxes were determined by searching for the consensus sequence 'GA**T**A**AT**GATA**A**TC**ATT**ATC' as used previously (Najimi *et al.*, 2008). Loci that retained all absolutely conserved nucleotides (shown in bold) and had greater than 50% identity to the overall consensus sequence were analyzed. Fur-boxes were identified in all strains, and several were conserved, including a surface-anchored protein from *Actinobacteria*. The actinobacterial surface-anchored protein is annotated based on the presence of the TIGR03769 HMM. These domains are typically located in two or more proteins encoded by a gene cluster, one of which is cell anchored. They are typically located proximal to the substrate-binding component of cognate ABC-transport systems, and it is thought they collectively act as a substrate-binding relay system. HMPREF0424_1241 appears to be encoded as part of a larger cluster of seven genes, four of which contain the TIGR03769 domain, two that are anchored by both N- and C-terminal transmembrane helices. The gene cluster also encompasses an ABC-transporter of unclear specificity. The ABC-transporter components each possess an anchored repeat-type ABC transporter domain (TIGR03770 – TIGR3772), while the substrate-binding protein additionally possesses the TIGR03769 domain. HMMs TIGR03770 –

TIGR3772 are described to engage strictly with the TIGR03769 domain. One fur-box, conserved between strain 409-05 and 594 (Table S7), maps immediately downstream of the isochorismatase, which likely has a role in siderophore production. The location of many of the Fur-box candidates, however, appeared cryptic occurring in agenic loci, often downstream of genes but a significant distance (always > 1kb) from the next potential ORF on the same DNA strand. A more focused effort on these regions also found no proximal pseudogene candidates (within 500 bp).
