# Supplementary figures and images for: Comparative Genomics of Gardnerella vaginalis Strains Reveals Substantial Differences in Metabolic and Virulence Potential
Source: PLoS One. 2010 Aug 26;5(8):e12411. doi: 10.1371/journal.pone.0012411 (PMC2928729; doi:10.1371/journal.pone.0012411)

Number of Orthologues

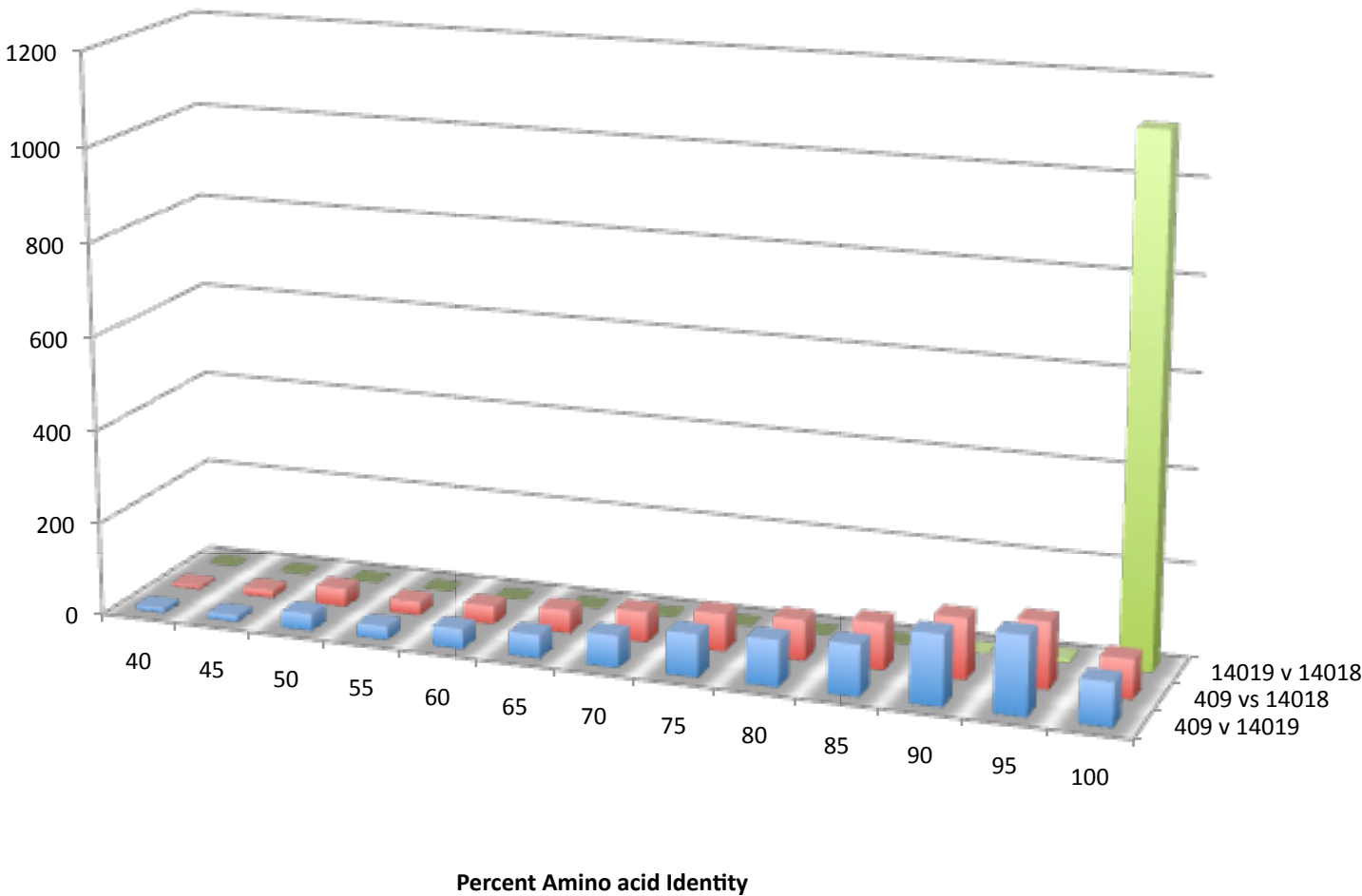

Supplement: Figure S1 — Orthologue protein sequence identity. Number of determined orthologues sharing each degree of amino acid sequence identity rounded to the nearest 5%. (0.07 MB PDF) [file pone.0012411.s003.pdf]

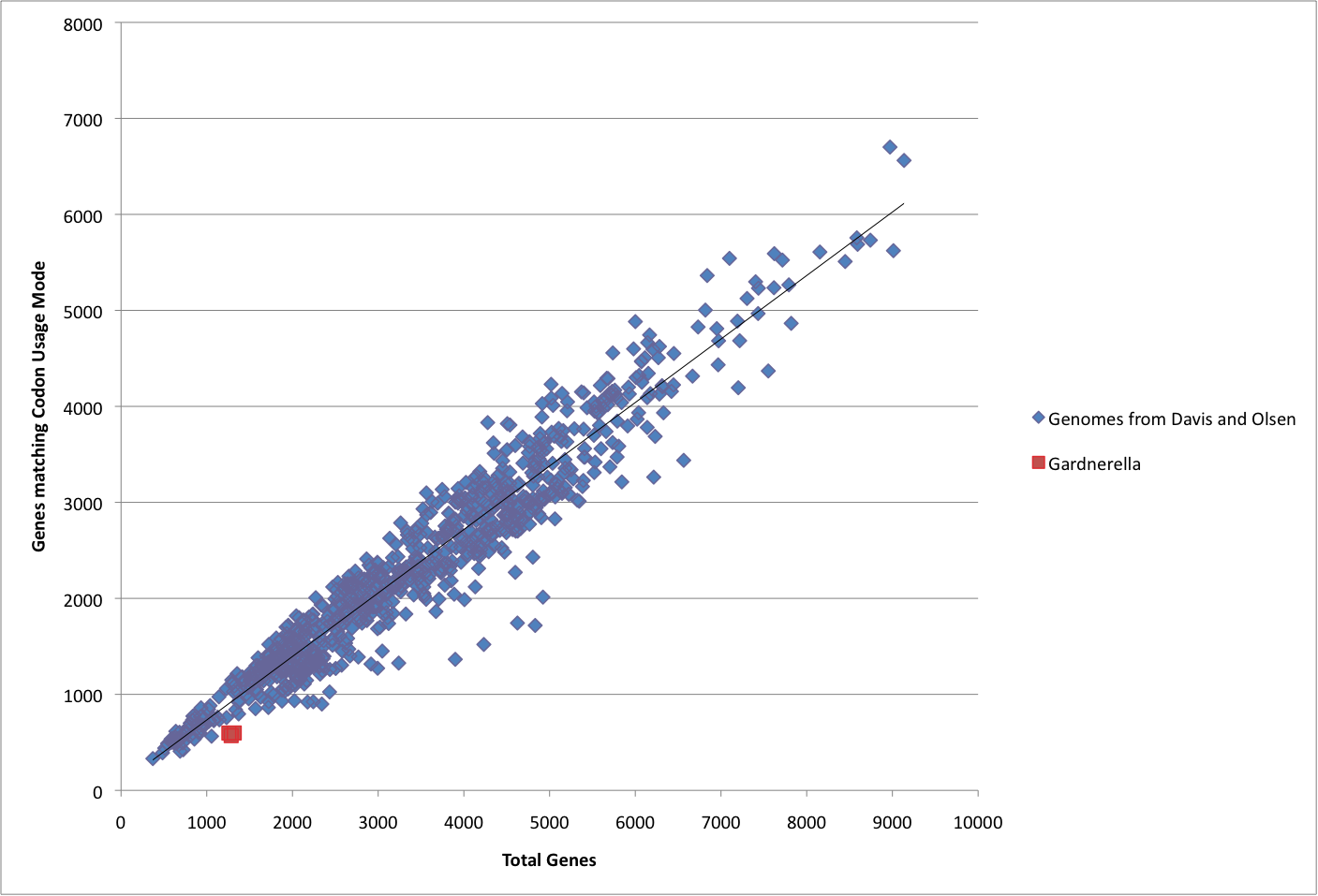

Supplement: Figure S2 — Proportion of native genes within Gardnerella vaginalis relative to other bacteria. The number of genes matching the modal codon usage relative to the number of genes within the genome of Gardnerella vaginalis (red) is ploted along with 923 other genomes (blue). (5.39 MB TIF) [file pone.0012411.s004.tif]
