# Supplementary material for: Comparative Genomics of Gardnerella vaginalis Strains Reveals Substantial Differences in Metabolic and Virulence Potential
Source: PLoS One. 2010 Aug 26;5(8):e12411. doi: 10.1371/journal.pone.0012411 (PMC2928729; doi:10.1371/journal.pone.0012411)
Supplement: Table S1 — Transfer RNA distribution. The number of tRNAs identified in each of the G. vaginalis genomes along with the anticodon recognized and amino acid transferred. (0.10 MB PDF) [file pone.0012411.s005.pdf]

Table S1 Transfer RNAs

tRNA #	tRNA gene 5′	tRNA gene 3′	tRNA Type	Anti-codon	Score
**Strain 409-05**					
1	171779	171851	Glu	CTC	55.69
2	171892	171963	Gln	CTG	53.54
3	211065	211146	Tyr	GTA	55
4	211148	211219	Thr	GGT	76.61
5	211224	211297	Met	CAT	76.14
6	217794	217880	Ser	GCT	55.55
7	246925	247010	Ser	CGA	59.44
8	333029	333113	Ser	TGA	60.04
9	390038	390110	Arg	CCG	79.79
10	415423	415496	Lys	CTT	80.83
11	417445	417517	Arg	CCT	72.01
12	449517	449588	Glu	TTC	61.7
13	450680	450753	Met	CAT	78.92
14	613221	613294	Leu	CAA	47.58
15	635799	635872	Pro	TGG	80.32
16	698358	698441	Leu	TAA	63.31
17	817174	817246	Gly	GCC	78.04
18	817275	817345	Cys	GCA	58.48
19	817376	817447	Val	GAC	73
20	817487	817559	Val	CAC	73.61
21	824252	824324	Gly	GCC	78.04
22	1040814	1040886	Asn	GTT	77.03
23	1577923	1577850	Ile	GAT	82.64
24	1577822	1577750	Ala	TGC	77.73
25	1571783	1571713	Gly	CCC	63.69
26	1550956	1550874	Leu	CAG	64.29
27	1394976	1394904	Trp	CCA	75.48
28	1347693	1347623	Gly	TCC	67.73
29	1327654	1327570	Ser	GGA	61.18
30	1235848	1235775	Asp	GTC	74.49
31	1235731	1235659	Phe	GAA	76.24
32	1129695	1129623	Arg	TCT	63.05
33	1110480	1110408	His	GTG	69.95
34	1091045	1090975	Gln	TTG	56.43
35	1083434	1083362	Ala	GGC	70.28
36	942939	942866	Arg	ACG	71.14
37	653340	653253	Leu	GAG	57.71
38	438697	438624	Val	TAC	77.34
39	368464	368391	Pro	CGG	77.42
40	314848	314776	Thr	CGT	71.28
41	314705	314625	Leu	TAG	66.62
42	179875	179803	Ala	CGC	73.79
43	108826	108753	Met	CAT	68.66
44	29836	29764	Lys	TTT	72.01
45	22414	22341	Thr	TGT	73.3
**Strain 317**					
1	40633	40706	Ile	GAT	82.64
2	40739	40811	Ala	TGC	77.73
3	46475	46545	Gly	CCC	64.76
4	253752	253824	Trp	CCA	75.48
5	375812	375885	Asp	GTC	74.49
6	375929	376001	Phe	GAA	76.24
7	377142	377213	Glu	TTC	61.7
8	452894	452966	Arg	CCT	72.01
9	562308	562381	Pro	TGG	80.32
10	654287	654360	Arg	ACG	71.14
11	697013	697085	Gly	GCC	78.04
12	697114	697184	Cys	GCA	58.48
13	697216	697287	Val	GAC	73
14	697328	697400	Val	CAC	73.61
15	750911	750994	Leu	TAA	64.71
16	1054968	1055041	Leu	CAA	47.58
17	1281398	1281471	Met	CAT	78.79
18	1338700	1338784	Ser	GGA	61.18
19	1350902	1350974	Thr	CGT	79.23
20	1351012	1351092	Leu	TAG	65.43
21	1533597	1533670	Met	CAT	68.66
22	1627129	1627201	Lys	TTT	72.01
23	1643652	1643725	Thr	TGT	73.38
24	1461681	1461609	Glu	CTC	52.82
25	1461568	1461497	Gln	CTG	53.54
26	1451459	1451378	Tyr	GTA	56.67
27	1451376	1451305	Thr	GGT	76.61
28	1451300	1451227	Met	CAT	76.14
29	1444807	1444721	Ser	GCT	55.77
30	1414785	1414700	Ser	CGA	60.18
31	1353334	1353264	Gly	TCC	73.56
32	1187735	1187651	Ser	TGA	60.04
33	1054718	1054646	Arg	TCT	66.71
34	1046866	1046794	Ala	GGC	70.28
35	922529	922457	His	GTG	71.46
36	910009	909939	Gln	TTG	56.43
37	886369	886297	Gly	GCC	78.04
38	756350	756278	Asn	GTT	77.03
39	552886	552800	Leu	GAG	58.92
40	540099	540027	Arg	CCG	79.26
41	520559	520486	Pro	CGG	70.17
42	481247	481174	Val	TAC	77.34
43	444467	444394	Lys	CTT	80.83
44	260852	260780	Ala	CGC	73.79
45	71049	70967	Leu	CAG	63.45

Strain 594					
1	2700	2785	Ser	CGA	60.18
2	44974	45044	Gly	CCC	64.76
3	283294	283367	Val	TAC	77.34
4	617904	617987	Leu	TAA	64.71
5	725612	725685	Arg	ACG	71.14
6	896495	896565	Gly	TCC	73.56
7	1102134	1102216	Leu	CAG	63.45
8	1223157	1223229	Arg	CCT	72.01
9	1326280	1326350	Gln	TTG	56.43
10	1348064	1348136	Gly	GCC	78.04
11	1426521	1426594	Pro	TGG	80.32
12	1577854	1577938	Ser	TGA	60.04
13	1505463	1505391	Arg	CCG	79.26
14	1485923	1485850	Pro	CGG	70.17
15	1417099	1417013	Leu	GAG	58.92
16	1253588	1253516	Glu	CTC	52.82
17	1253475	1253404	Gln	CTG	53.54
18	1243366	1243285	Tyr	GTA	56.67
19	1243283	1243212	Thr	GGT	76.61
20	1243207	1243134	Met	CAT	76.14
21	1214731	1214658	Lys	CTT	80.83
22	1003419	1003347	Ala	CGC	73.79
23	911129	911045	Ser	GGA	61.18
24	898927	898855	Thr	CGT	79.23
25	898817	898737	Leu	TAG	65.43
26	885573	885500	Asp	GTC	74.49
27	885456	885384	Phe	GAA	76.24
28	884243	884172	Glu	TTC	61.7
29	807731	807659	His	GTG	71.46
30	764685	764613	Lys	TTT	72.01
31	694498	694426	Ala	GGC	70.28
32	623343	623271	Asn	GTT	77.03
33	406647	406574	Thr	TGT	73.38
34	247357	247284	Met	CAT	78.79
