# Supplementary material for: Comparative Genomics of Gardnerella vaginalis Strains Reveals Substantial Differences in Metabolic and Virulence Potential
Source: PLoS One. 2010 Aug 26;5(8):e12411. doi: 10.1371/journal.pone.0012411 (PMC2928729; doi:10.1371/journal.pone.0012411)
Supplement: Table S2 — Genes potentially involved in competence. Genes identified within the G. vaginalisgenomes whose annotated roles have been shown to promote competence in other bacteria. (0.06 MB PDF) [file pone.0012411.s006.pdf]

Table S2 Genes involved in competence

Locus tag			Product	Orthology (% ID)
409-05	317	594		a-b / b-c / c-a
HMPREF0424_0942	HMPREF0421_20735	816	Competence protein, ComEC	42 / 100 / 49
HMPREF0424_0943	n/a	n/a	Competence protein, ComEA	- / - / -
n/a	HMPREF0421_20734	815	Competence protein, ComEA	- / 100 / -
HMPREF0424_1021	HMPREF0421_20866	122	RecA	92 / 100 / 92
HMPREF0424_1022	HMPREF0421_20868	124	CinA-family competence protein	48 / 100 / 48

n/a – Orthologue was not identified within the genome
