# Supplementary material for: Comparative Genomics of Gardnerella vaginalis Strains Reveals Substantial Differences in Metabolic and Virulence Potential
Source: PLoS One. 2010 Aug 26;5(8):e12411. doi: 10.1371/journal.pone.0012411 (PMC2928729; doi:10.1371/journal.pone.0012411)
Supplement: Table S4 — Genes characteristic of mobile elements. Genes identified within each G. vaginalis genome that are likely to have been components of mobile elements. (0.06 MB PDF) [file pone.0012411.s008.pdf]

Table S4 Genes characteristic of mobile elements

Locus Tag			Product	Orthology (% ID)
409-05 (a)	317 (b)	594 (c)		a-b / b-c / c-a
n/a	HMPREF0421_20010	1192	Phage integrase/site specific recombinase	- / 100 / -
n/a	HMPREF0421_20030	433	IS3509a-family transposase	- / 100 / -
HMPREF0424_0509	n/a	n/a	IS150-family transposase	- / - / -
HMPREF0424_0698	HMPREF0421_20610	n/a	HK97-family phage prohead protease	76 / - / -
HMPREF0424_0850	HMPREF0421_20580	643	GepA family prophage protein	41 / 100 / 41
HMPREF0424_0875	n/a	n/a	Phage capsid protein	- / - / -
HMPREF0424_0956	n/a	n/a	Phage integrase/site specific recombinase	- / - / -
HMPREF0424_0994	n/a	n/a	TP901-family phage tail tape measure protein	- / - / -
n/a	HMPREF0421_20612	1028	Prophage ps3 protein	- / 100 / -
n/a	HMPREF0421_20580	643	Phage-associated protein	- / 100 / -
