# Supplementary material for: Comparative Genomics of Gardnerella vaginalis Strains Reveals Substantial Differences in Metabolic and Virulence Potential
Source: PLoS One. 2010 Aug 26;5(8):e12411. doi: 10.1371/journal.pone.0012411 (PMC2928729; doi:10.1371/journal.pone.0012411)
Supplement: Table S5 — Nitrogen metabolism genes. Genes identified within the G. vaginalis genomes that appear to encode functions important for the utilization of various nitrogen sources. (0.08 MB PDF) [file pone.0012411.s009.pdf]

Table S5 Nitrogen Metabolism Genes

Locus Tag			Enzyme	Orthology (% ID)
409-05 (a)	317 (b)	594 (c)		a-b / b-c / c-a
**Ammonia utilization**				
HMPREF0424_0073	86	97	Amt-family ammonia transporter	72 / 100 / 72
HMPREF0424_0474	n/a	n/a	Asp-synthase (glutamine-hydrolyzing)	- / - / -
HMPREF0424_0930	726	1147	Glutamate-ammonia ligase	78 / 100 / 78
**Amino acid utilization**				
HMPREF0424_0111	n/a	n/a	Aspartate transaminase	- / - / -
HMPREF0424_1220	312	n/a	Aspartate transaminase	85 / - / -
HMPREF0424_0134	27	440	Amino acid permease	87 / / 87
HMPREF0424_0241	1255	n/a	Amino acid permease	92 / - / -
HMPREF0424_0196	1274	497	LIVCS-family branched chain amino acid symporter	92 / 100 / 92
HMPREF0424_0235	1258	n/a	Histidinol phosphate transaminase	83 / - / -
HMPREF0424_0547	1001	880	DAACS-family dicarboxylate/amino acid:sodium symporter	95 / 100 / 95
HMPREF0424_0786	n/a	n/a	Glutamine-specific ABC-transport system	- / - / -
HMPREF0424_0787	802	n/a		87 / - / -
HMPREF0424_0788	801	n/a		90 / - / -
HMPREF0424_0789	800	n/a		87 / - / -
HMPREF0424_0858	703	805	Branched chain amino acid transaminase	96 / 100 / 96
n/a	366	1159	Amino acid-specific ABC transporter	- / 100 / -
n/a	365	1158		- / 100 / -
n/a	364	1157		- / 100 / -
HMPREF0424_0954	705	n/a	Lysine-specific permease	86 / - / -
**Protein/Peptide utilization**				
HMPREF0424_0092	69	n/a	Clp-family ATP-dependent endopeptidase	99 / - / -
HMPREF0424_0878	n/a	n/a	Clp-family ATP-dependent endopeptidase	- / - / -
HMPREF0424_0879	622	61	Clp-family ATP-dependent endopeptidase	93 / 100 / 93
HMPREF0424_0302	380	522	Oligopeptidase	64 / 100 / 64
HMPREF0424_0436	471	n/a	Dipeptide-specific ABC transporter	68 / - / -
HMPREF0424_0437	472	n/a		69 / - / -
HMPREF0424_0438	473	n/a		71 / - / -
HMPREF0424_0439	n/a	n/a		- / - / -
HMPREF0424_1149	1148	1048	Membrane-bound alanyl aminopeptidase	95 / 100 / 95
HMPREF0424_0563	976	n/a	C15-family pyrrolidone-carboxylate peptidase	95 / - / -
n/a	325	281	C15-family pyrrolidone-carboxylate peptidase	- / 100 / -
n/a	n/a	983	C15-family pyrrolidone-carboxylate peptidase	- / - / -
HMPREF0424_1262	261	115	C69-family dipeptidase	95 / 100 / 95
n/a	n/a	56	M13-family peptidase	- / - / -
HMPREF0424_0408	536	692	M20-family peptidase	89 / 100 / 89
HMPREF0424_0463	1147	1049	M24-family peptidase	89 / 99 / 88
HMPREF0424_0007	154	876	M48-family peptidase	53 / 100 / 53
HMPREF0424_1074	905	n/a	Methionine aminopeptidase	100 / - / -
HMPREF0424_0447	482	479	S16-family peptidase	56 / 100 / 56
HMPREF0424_0025	139	1071	S54-family peptidase	65 / 100 / 65
HMPREF0424_0423	466	n/a	Cell wall-associated serine proteinase	42 / - / -
n/a	n/a	464	Cell wall-associated serine proteinase	- / - / -
