# Supplementary material for: Comparative Genomics of Gardnerella vaginalis Strains Reveals Substantial Differences in Metabolic and Virulence Potential
Source: PLoS One. 2010 Aug 26;5(8):e12411. doi: 10.1371/journal.pone.0012411 (PMC2928729; doi:10.1371/journal.pone.0012411)
Supplement: Table S6 — Strain variable enzymes. Genes identified from one or more of the G. vaginalis genomes that are predicted to encode enzymes whose functions do not appear to be encoded for in the other strain(s). (0.09 MB PDF) [file pone.0012411.s010.pdf]

Table S6 Strain variable enzymes

Process	EC Number	Functions
**409-05 only[1]**		
Amino acid metabolism	2.3.1.46	Homoserine O-succinyltransferase (HMPREF0424_0141)
	2.5.1.49	O-acetylhomoserine aminocarboxypropyltransferase (HMPREF0424_0640)
	4.2.1.20	Tryptophan synthase, beta subunit (HMPREF0424_1349)
	5.1.1.13	Aspartate racemase (HMPREF0424_0465)
Amino acid metabolism / Nitrogen metabolism	6.3.5.4	Asparagine synthase (glutamine-hydrolyzing) (HMPREF0424_0474)
Folate biosynthesis	2.5.1.15	Dihydropteroate synthase (HMPREF0424_0665)
	2.7.6.3	2-amino-4-hydroxy-6- hydroxymethyldihydropteridine diphosphokinase (HMPREF0424_0619)
PTS transport	2.7.3.9	Phosphoenolpyruvate phosphotransferase system enzyme I (HMPREF0424_1268)
Nucleic acid metabolism	3.1.21.-	LlaJI-family restriction endonuclease (HMPREF0424_0537)
	3.5.2.5	Allantoinase (HMPREF0424_0775)
	3.5.3.9	Allantoate amidohydrolase (HMPREF0424_0773)
	3.5.4.-	Cytidine/deoxycytidylate deaminase (HMPREF0424_0498)
Glycosylase	3.2.2.24	ADP-ribosylglycohydrolase (HMPREF0424_0289)
**317 only[1]**		
Protein regulation	2.7.11.-	Serine/threonine protein kinase (HMPREF0421_20141)
Nucleic acid metabolism	3.1.4.-	Rne/Rng family ribonuclease (HMPREF0421_20320)
Glycan degradation	3.2.1.18	Sialidase (HMPREF0421_20186)
Toluene and xylene degradation	5.1.2.2	Mandelate racemase/muconate lactonizing enzyme C-terminal domain protein (HMPREF0421_20695)
Glycolysis / Gluconeogenesis	5.1.3.3	Aldose 1-epimerase (HMPREF0421_21170)
**594 only**		
Aminosugar metabolism	2.7.7.23	N-acetylglucosamine diphosphorylase (ORF 865)
Glycogen/starch metabolism	5.4.99.16	Trehalose synthase (ORF 883)
Nucleic acid metabolism	5.99.1.-	Topoisomerase IV subunit B (ORF 1053)
**409-05 and 317[1]**		
Lipid metabolism	2.3.1.157	UDP-N-acetylglucosamine diphosphorylase/glucosamine-1-phosphate N-acetyltransferase (HMPREF0424_1013 & HMPREF0421_20975)
Fatty acid biosynthesis	2.3.1.85	Fatty acid synthase (HMPREF0424_1197 & HMPREF0421_21228)
Amino acid metabolism	2.6.1.1	Aspartate transaminase (HMPREF0424_1220 & HMPREF0421_20325)
Amino acid metabolism / Drug metabolism	2.4.2.3	Pnp/Udp-family phosphorylase (HMPREF0424_0551 & HMPREF0421_21030)
Nicotinate & Nicotinamide metabolism	2.7.1.23	NAD(+)/NADH kinase (HMPREF0424_0680 & HMPREF0421_20594)
Vitamin metabolism	3.6.1.9	Type I phosphodiesterase/nucleotide pyrophosphatase (HMPREF0424_0700 & HMPREF0421_20620)
Protein modification	3.4.24.71	Endothelin-converting enzyme (HMPREF0424_1076 & HMPREF0421_20934)
Amino acid metabolism	3.5.1.4	Amidohydrolase (HMPREF0424_0452 & HMPREF0421_20509)
Lipid metabolism	3.1.1.3	Triacylglycerol lipase (HMPREF0424_0004 & HMPREF0421_20172)
Glycolysis / Gluconeogenesis	4.1.2.13	Fructose-bisphosphate aldolase class II (HMPREF0424_0114 & HMPREF0421_20056)
	4.2.1.11	Enolase (HMPREF0424_0662 & HMPREF0421_20718)
**317 and 594**		
Amino acid metabolism	2.3.1.3	Serine acetyltransferase (HMPREF0421_21032 & ORF 878)
	2.7.2.4	Aspartate kinase (HMPREF0421_20041 & ORF 446)
	2.7.2.4	Aspartate kinase (HMPREF0421_20042 & ORF 447)
	3.3.1.1	S-adenosyl-L-homocysteine hydrolase (HMPREF0421_20382 & ORF 512)
	3.5.99.2	TENA/THI-4 protein (HMPREF0421_21010 & ORF 986)
	4.2.1.52	Dihydrodipicolinate synthase (HMPREF0421_20180 & ORF 1097)
	4.2.1.52	Dihydrodipicolinate synthase (HMPREF0421_20691 & ORF 1012)
Carbohydrate metabolism	3.2.1.135	Neopullulanase (HMPREF0421_20921 & ORF 47)
	3.2.1.2	Glycoside hydrolase family 31 (HMPREF0421_20452 & ORF 423)
	5.1.3.13	Fused dTDP-4-keto-L-rhamnose reductase and dTDP-4-keto-6-deoxyglucose-3,5-epimerase enzyme (HMPREF0421_20400 & ORF 530)
	2.4.1.230	Kojibiose phosphorylase (HMPREF0421_21250 & ORF 1088)
	5.1.3.4	L-Ribulose-5-phosphate 4-epimerase (HMPREF0421_20373 & ORF 1154)
	5.3.1.4	L-Arabinose isomerase (HMPREF0421_20374 & ORF 1155)
	2.7.1.6	Galactokinase (HMPREF0421_21182 & ORF 1193)
	3.2.1.23	Beta-galactosidase (HMPREF0421_20100 & ORF 1134)
	3.2.1.23	Beta-galactosidase (HMPREF0421_21246 & ORF 1092)
	2.7.1.2	Glucokinase (HMPREF0421_20174 & ORF 900)
	2.7.1.2	Glucokinase (HMPREF0421_20897 & ORF 830)
Glycan degradation	3.2.1.51	α-L-fucosidase (HMPREF0421_20101 & ORF 1135)
Glycogen/starch metabolism	5.4.2.6	β-phosphoglucomutase (HMPREF0421_21248 & ORF 1090)
N-acetylglucosamine metabolism	3.5.1.25	N-acetylglucosamine-6-phosphate deacetylase (HMPREF0421_20182 & ORF 1099)
	3.5.99.6	Glucosamine-6-phosphate deaminase (HMPREF0421_20181 & ORF 1098)
	5.1.3.8	N-acyl-D-glucosamine 2-epimerase (HMPREF0421_20908 & ORF 1042)
	5.1.3.9	N-acylglucosamine-6-phosphate 2-epimerase (HMPREF0421_20175 & ORF 899)
Xylose metabolism	2.7.1.16	Ribulokinase (HMPREF0421_20372 & ORF 1153)
Pentose phosphate pathway	2.7.1.12	Gluconokinase (HMPREF0421_20297 & ORF 1005)
Nucleic acid metabolism	3.5.4.28	S-adenosylhomocysteine deaminase (HMPREF0421_20381 & ORF 511)
Ascorbate biosynthesis	1.1.1.274	2,5-didehydrogluconate reductase (HMPREF0421_20375 & ORF 1156)
Nicotinate & Nicotinamide metabolism	2.7.7.1	Galactose-1-phosphate uridylyltransferase (HMPREF0421_21097 & ORF 765)
	2.7.7.1	Galactose-1-phosphate uridylyltransferase (HMPREF0421_21183 & ORF 93)
Protein modification	2.7.1.95	Aminoglycoside phosphotransferase (HMPREF0421_20507 & ORF 1079)
Bile acid metabolism	3.5.1.24	Choloylglycine hydrolase (HMPREF0421_21234 & ORF 145)
Glyoxylate & dicarboxylate metabolism	1.1.1.77	Lactaldehyde reductase (HMPREF0421_20697 & ORF 18)
Terpenoid biosynthesis	2.5.1.-	1,4-dihydroxy-2-naphthoate octaprenyltransferase (HMPREF0421_20560 & ORF 622)
	2.7.7.24	Glucose-1-phosphate thymidylyltransferase (HMPREF0421_20399 & ORF 529)

[1] These enzymes may also be present in the missing segments of the strain 594 genome.
Enzymes with ambiguous EC numbers are not reported
