# Supplementary material for: Comparative Genomics of Gardnerella vaginalis Strains Reveals Substantial Differences in Metabolic and Virulence Potential
Source: PLoS One. 2010 Aug 26;5(8):e12411. doi: 10.1371/journal.pone.0012411 (PMC2928729; doi:10.1371/journal.pone.0012411)
Supplement: Table S7 — Carbohydrate metabolism genes. Genes present in one or more of the G. vaginalis genomes that appear to encode functions important to the utilization of various carbohydrates. (0.09 MB PDF) [file pone.0012411.s011.pdf]

Table S7 Carbohydrate metabolism genes

Locus Tag			Enzyme	Orthology (% ID)
409-05 (a)	317 (b)	594 (c)		a-b / b-c / c-a
**N-acetylglucosamine utilization**				
n/a	HMPREF0421_20182	1099	N-acetylglucosamine-6-phosphate deacetylase	- / 100 / -
n/a	HMPREF0421_20181	1098	Glucosamine-6-phosphate deaminase	- / 100 / -
n/a	HMPREF0421_20182	21	N-acetylglucosamine kinase	- / 100 / -
**Arabinose utilization**				
n/a	HMPREF0421_20377	1155	L-arabinose isomerase	- / 100 / -
n/a	HMPREF0421_20376	1154	L-ribulose-5-phosphate 4-epimerase	- / 100 / -
**Fructose utilization**				
HMPREF0424_0462	HMPREF0421_21188	1050	Fructokinase	84 / 100 / 84
**Galactose utilization**				
n/a	HMPREF0421_21095	1101	Galactose-1-phosphate uridylyltransferase	- / 100 / -
n/a	HMPREF0421_20183	93		- / 100 / -
n/a	HMPREF0421_20182	92	Galactokinase	- / 100 / -
**Glucose utilization**				
HMPREF0424_0343	HMPREF0421_21223	197	Glucose-6-phosphate isomerase	98 / 100 / 98
HMPREF0424_0820	HMPREF0421_20797	14	Polyphosphate glucokinase	97 / 100 / 97
n/a	HMPREF0421_20897	830	Glucokinase	- / 100 / -
**Glycogen/Starch utilization**				
HMPREF0424_0023	HMPREF0421_20150	1073	Glycogen phosphorylase	99 / 100 / 99
HMPREF0424_0619	HMPREF0421_20568	630	Glycogen debranching enzyme	93 / 100 / 93
HMPREF0424_0298	HMPREF0421_20391	518	Phosphoglucomutase	99 / 100 / 99
HMPREF0424_0609	n/a	n/a	α-Amylase	- / - / -
HMPREF0424_0610	HMPREF0421_21227	1171		56 / 100 / 56
HMPREF0424_1294	HMPREF0421_20243	n/a		85 / - / -
HMPREF0424_1313	HMPREF0421_20224	232		98 / 100 / 98
HMPREF0424_1317	n/a	n/a		- / - / -
n/a	HMPREF0421_20825	n/a	UTP-glucose-1-phosphate uridylyltransferase	- / - / -
HMPREF0424_0790	HMPREF0421_20826	1233		87 / 100 / 89
HMPREF0424_1332	HMPREF0421_20204	n/a	Oligo-1,6-glucosidase	85 / - / -
n/a	n/a	883	Trehelose synthase	- / - / -
**Xylose utilization**				
		848	Xylulokinase	
**Glycolysis**				
n/a	HMPREF0421_21170	n/a	Aldose-1-epimerase	- / - / -
n/a	n/a	n/a	Phosphofructokinase	- / - / -
HMPREF0424_0114	HMPREF0421_20056	n/a	Fructose-bisphosphate aldolase class II	82 / - / -
HMPREF0424_0471	HMPREF0421_21168	77	Glyceraldehyde-3-P dehydrogenase	98 / 100 / 98
HMPREF0424_0908	HMPREF0421_20767	586	Phosphoglycerate kinase	98 / 100 / 98
HMPREF0424_0199	HMPREF0421_21309	491	Phosphoglycerate mutase	78 / 100 / 78
HMPREF0424_0371	HMPREF0421_21094	n/a		84 / - / -
HMPREF0424_1011	HMPREF0421_20973	n/a		90 / - / -
HMPREF0424_1307	HMPREF0421_20231	240		68 / 100 / 68
n/a	n/a	766		- / - / -
n/a	n/a	767		- / - / -
HMPREF0424_0394	HMPREF0421_20538	678	2,3-Bisphosphoglycerate-dependent mutase	100 / 100 / 100
HMPREF0424_0662	HMPREF0421_20718	n/a	Enolase	99 / - / -
HMPREF0424_0607	HMPREF0421_20976	n/a	Pyruvate kinase	95 / - / -
**Pentose phosphate pathway**				
HMPREF0424_0185	HMPREF0421_21326	506	Glucose-6-phosphate dehydrogenase	94 / 100 / 94
HMPREF0424_0183	HMPREF0421_21328	509	6-Phosphogluconolactonase	81 / 100 / 81
HMPREF0424_0180	HMPREF0421_21329	n/a	6-Phosphogluconate dehydrogenase	98 / - / -
n/a	n/a	n/a	Ribulose-5-phosphate isomerase	
HMPREF0424_0803	HMPREF0421_20101	786 / 890	Ribulose-5-phosphate 3-epimerase	93 / 100 / 93
HMPREF0424_0902	HMPREF0421_20790	8	Transketolase	99 / 100 / 99
HMPREF0424_0903	HMPREF0421_20791	7	Transaldolase	99 / 100 / 99
n/a	HMPREF0421_20174	900	Gluconokinase	- / 100 / -
**TCA cycle**				
n/a	n/a	n/a	Citrate synthase	- / - / -
n/a	n/a	n/a	Aconitase	- / - / -
n/a	n/a	n/a	Isocitrate dehydrogenase	- / - / -
n/a	n/a	n/a	α-Ketogluterate dehydrogenase	- / - / -
n/a	n/a	n/a	Succinyl-CoA synthase	- / - / -
HMPREF0424_0636	HMPREF0421_20564	626	Succinate dehydrogenase	62 / 100 / 63
HMPREF0424_0867	HMPREF0421_20964	37	Succinate dehydrogenase	62 / 100 / 62
n/a	n/a	n/a	Fumarase	- / - / -
HMPREF0424_0663	HMPREF0421_20719	n/a	Malate dehydrogenase	92 / - / -

n/a – indicates enzyme was not identified within the available genome sequence
