# Supplementary material for: Comparative Genomics of Gardnerella vaginalis Strains Reveals Substantial Differences in Metabolic and Virulence Potential
Source: PLoS One. 2010 Aug 26;5(8):e12411. doi: 10.1371/journal.pone.0012411 (PMC2928729; doi:10.1371/journal.pone.0012411)
Supplement: Table S8 — Genes potentially important to effector protein translocation. Genes identified within each G. vaginalis strain that are potentially important to effector protein translocation. (0.06 MB PDF) [file pone.0012411.s012.pdf]

Table S8 Genes potentially involved in effector protein translocation

409-05 (a)	317 (b)	594 (c)	Product	Orthology (% ID) a-b / b-c / c-a
HMPREF0424_0063	HMPREF0421_20109	1168	G5/lytic transglycosylase-domain protein	64 / 100 / 61
HMPREF0424_0355	HMPREF0421_20417	1242	G5/lytic transglycosylase-domain protein	75 / 100 / 75
HMPREF0424_0120	HMPREF0421_20050	455	Type II/IV secretion system protein (VirB11-like)	67 / 100 / 67
HMPREF0424_0685	HMPREF0421_20599	n/a	Type II secretion system preprotein translocase	91 / - / -
