# Supplementary material for: Comparative Genomics of Gardnerella vaginalis Strains Reveals Substantial Differences in Metabolic and Virulence Potential
Source: PLoS One. 2010 Aug 26;5(8):e12411. doi: 10.1371/journal.pone.0012411 (PMC2928729; doi:10.1371/journal.pone.0012411)
Supplement: Table S9 — Sortase enzymes and proteins cantaining LPxTG motifs. All sortase encoding enzymes and proteins carrying LPxTG motifs within the genomes of the G. vaginalis strains. Note LPxTG motif-containing proteins are typically attached to the cell surface by sortase enzymes. (0.08 MB PDF) [file pone.0012411.s013.pdf]

Table S9 Sortase enzymes and proteins containing LPxTG motifs

Locus tag			Product	Orthology (% ID) a-b / b-c / c-a
409-05	317	594		
HMPREF0424_0029	HMPREF0421_20143	399	Sortase D family protein	60 / 100 / 60
HMPREF0424_0422	HMPREF0421_21113	750	Sortase family protein	69 / 100 / 69
HMPREF0424_0427	n/a	n/a	Sortase C family protein	- / - / -
HMPREF0424_1025	HMPREF0421_20501	n/a	Sortase C family protein	83 / - / -
HMPREF0424_1161	HMPREF0421_21203	1122	Sortase C family protein	88 / 100 / 88
HMPREF0424_1214	n/a	n/a	Sortase C family protein	- / - / -
n/a	n/a	1085	Sortase C family protein	- / - / -
**Proteins containing LPxTG motifs (Sortase substrates)**				
HMPREF0424_0024	HMPREF0421_20149	1072	Conserved hypothetical protein	88 / 100 / 88
HMPREF0424_0307	HMPREF0421_21238[2,3]	140[2]	LPxTG-domain protein	85 / 100 / 85
HMPREF0424_0418	n/a	n/a	LPxTG-domain protein	- / - / -
HMPREF0424_0420	n/a	n/a	LPxTG-domain protein	- / - / -
HMPREF0424_0426	n/a	n/a	LPxTG-domain protein	- / - / -
HMPREF0424_0541	n/a	n/a	LPxTG-domain protein	- / - / -
HMPREF0424_0545	HMPREF0421_20447[2]	427[2]	GA-module protein	50 / 100 / 50
HMPREF0424_0894	n/a	n/a	LPxTG-domain protein	- / - / -
HMPREF0424_1026	HMPREF0421_21115	749	Type-I fimbrial major subunit precursor	61 / 100 / 61
HMPREF0424_1062	n/a	n/a	LPxTG-domain protein	- / - / -
HMPREF0424_1164	HMPREF0421_21204	1121	Type-II fimbrial major subunit precursor	43 / 100 / 44
HMPREF0424_1196	HMPREF0421_21226[2]	n/a	Rib-protein	76 / - / -
HMPREF0424_1208	n/a	n/a	LPxTG-domain protein	- / - / -
HMPREF0424_1215	n/a	n/a	LPxTG-domain protein	- / - / -
HMPREF0424_1216	n/a	n/a	LPxTG-domain protein	- / - / -
n/a	HMPREF0421_20012	1190	LPxTG-domain protein	- / 100 / -
n/a	HMPREF0421_20167	930	LPxTG-domain protein	- / 100 / -
n/a	HMPREF0421_20170	n/a	LPxTG-domain protein	- / - / -
n/a	HMPREF0421_20499	1000	Conserved hypothetical protein	- / 100 / -
n/a	HMPREF0421_20500	n/a	Type-I fimbrial major subunit precursor	- / - / -
n/a	HMPREF0421_20608	n/a	Conserved hypothetical protein	- / - / -
n/a	HMPREF0421_20611	1027	LPxTG-domain protein	- / 100 / -
n/a	HMPREF0421_20617	n/a	LPxTG-domain protein	- / - / -
n/a	HMPREF0421_20618	997	Conserved hypothetical protein	- / 100 / -
n/a	HMPREF0421_20949	267	LPxTG-domain protein	- / 100 / -
n/a	n/a	999	Conserved hypothetical protein	- / - / -
n/a	n/a	1023	Conserved hypothetical protein	- / - / -
n/a	n/a	1184	LPxTG-domain protein	- / - / -

n/a - indicates protein was not identified within the genome
