# Supplementary material for: Comparative Genomics of Gardnerella vaginalis Strains Reveals Substantial Differences in Metabolic and Virulence Potential
Source: PLoS One. 2010 Aug 26;5(8):e12411. doi: 10.1371/journal.pone.0012411 (PMC2928729; doi:10.1371/journal.pone.0012411)
Supplement: Table S10 — Potential FUR-regulatory sequences. All identified FUR-box sequences identified within each G. vaginalis strain along with proximal downstream genes, which may be regulated by the FUR-family transcriptional regulator. (0.08 MB PDF) [file pone.0012411.s014.pdf]

Table S10 Potential Fur-regulatory sequences

3' - Proximal gene		Fur-box sequence	Identities
Locus Tag	Product		
	**409-05**		
HMPREF0421_0142[3]	ATP synthase F0, subunit A	CGTAATCGTTATGATTATT	12/19
HMPREF0424_1188	Phosphotransferase-family protein	ACTAATAATAATTATTTTT	13/19
HMPREF0424_1233	D-ala D-ala ligase-like protein	AATAATTAGAATAATTCAA	12/19
HMPREF0424_1241	Actinobacterial surface-anchored protein	ATTAATGAGAATCATTATT	15/19
Unclear[1]	Unclear	AATTATGAGAATAATTTTC	13/19
Unclear[1]	Unclear	CCTAATGGGAATAATTGCC	12/19
Unclear[1]	Unclear	CGTAATCGTTATTATTTGA	10/19
Unclear[1]	Unclear	GTTAATGAGAATCATTATC	17/19
Unclear[1]	Unclear	TTTTATTAGAATAATTACT	11/19
Unclear[1,4]	Unclear	AATAATAATAATGATTCTC	15/19
	**317**		
HMPREF0421_20009[3]	ATP synthase F0, subunit A	CGTAATCGTTATGATTATT	12/19
HMPREF0421_20303	Actinobacterial surface-anchored protein	ATTAATGAGAATCATTATT	15/19
HMPREF0421_20307	Hypothetical protein	GTTTATGAGAATGATTCTC	14/19
HMPREF0421_20603[3]	Kinase	TTTAATAAGAATGATTTAA	11/19
HMPREF0421_20711[3]	ABC transporter, permease protein	AGTAATAGTAATGATTTTT	12/19
HMPREF0421_20763[3]	Conserved hypothetical protein	AATTATAAGAATAATTGCA	11/19
HMPREF0421_20840	Peroxiredoxin	CTTAATGATAATAATTATC	16/19
HMPREF0421_20841	Alcohol dehydrogenase	GATAATTATTATCATTAAG	15/19
HMPREF0421_20864[3]	Glutamate-5-semialdehyde dehydrogenase	CGTAATGGGAATTATTTAC	12/19
HMPREF0421_20879	Conserved hypothetical protein	TTTTATCATTATAATTGAT	10/19
HMPREF0421_21091	Conserved hypothetical protein	GATTATAAGAATGATTGCA	12/19
HMPREF0421_21130[3]	Site-specific tyrosine recombinase, *xerC*	CGTAATAATAATGATTTAT	12/19
HMPREF0421_21326	Glucose-6-phosphate dehydrogenase	CGTAATAGTTATTATTGCA	10/19
HMPREF0421_21337	ATPase	TATTATAATTATGATTATA	13/19
Unclear[1]	Unclear	CCTAATGGGAATAATTGCC	12/19
Unclear[1]	Unclear	ACTAATTATAATAATTTCT	12/19
Unclear[1,2]	Unclear	CATTATTAGAATAATTTGC	12/19
Unclear[1,2]	Unclear	AATAATCGTAATTATTTAA	12/19
Unclear[1]	Unclear	AGTTATAAGAATTATTACG	11/19
Unclear[1]	Unclear	TTTTATAAGAATAATTAAA	11/19
Unclear[1]	Unclear	TGTAATCATTATGATTTAC	12/19
Unclear[1]	Unclear	AGTAATTATAATTATTTAA	12/19
	**594**		
55	Methionine aminopeptidase	TATTATTATTATCATTGTT	13/19
127[3]	Ftsk/SpoIIIE-family protein	TGTAATGATTATTATTGCT	12/19
134[3]	Arylsulfatase	AATAATGGTTATGATTGTG	13/19
544[3]	Glutamate-5-semialdehyde dehydrogenase	CGTAATGGGAATTATTTAC	12/19
566	Alcohol dehydrogenase	GATAATTATTATCATTAAG	15/19
567	Peroxiredoxin	CTTAATGATAATAATTATC	16/19
733[3]	Site-specific tyrosine recombinase, *xerC*	CGTAATAATAATGATTTAT	12/19
772	Conserved hypothetical protein	GATTATAAGAATGATTGCA	12/19
939[3]	ATP synthase F0, subunit A	CGTAATCGTTATGATTATT	12/19
1057[3]	Kinase	TTTAATAAGAATGATTTAA	11/19
1222	Actinobacterial surface-anchored protein	ATTAATGAGAATCATTATT	15/19
1259[3]	ABC transporter, permease protein	AGTAATAGTAATGATTTTT	12/19
1276	ATPase	TATTATAATTATGATTATA	13/19
Unclear[1,4]	Unclear	AATAATAATAATGATTCTC	15/19
Unclear[1]	Unclear	AGTAATTATAATTATTTAA	12/19
Unclear[1]	Unclear	TTTTATAAGAATAATTAAA	11/19
Unclear[1]	Unclear	TGTAATCATTATGATTTAC	12/19
Unclear[1]	Unclear	CATTATTAGAATAATTTGC	12/19
	**Consensus sequence**	GA**T**A**AT**GATAA**T**CA**TT**ATC	

[1] Fur-box located in region devoid of proximal gene candidates (within 1 kb) on same DNA strand. [2] A potential uncalled hypothetical gene is found downstream of Fur-box but overlaps larger ORF with functional assignment [3] Fur box located within gene sequence [4] Downstream of isochorismatase
